# Supplementary material for: Modulation of APLNR Signaling Is Required during the Development and Maintenance of the Hematopoietic System
Source: Stem Cell Reports. 2021 Mar 4;16(4):727–40. doi: 10.1016/j.stemcr.2021.02.003 (PMC8072025; doi:10.1016/j.stemcr.2021.02.003)
Supplement: Document S1. Figures S1–S6 [file mmc1.pdf]

Stem Cell Reports, Volume *16*

Supplemental Information

Modulation of APLNR Signaling Is Required during the Development and Maintenance of the Hematopoietic System

Melany Jackson, Antonella Fidanza, A. Helen Taylor, Stanislav Rybtsov, Richard Axton, Maria Kydonaki, Stephen Meek, Tom Burdon, Alexander Medvinsky, and Lesley M. Forrester

Jackson et al Supplementary Figures

Supplementary Figure S1. Production of *Aplnr*-tdTomato reporter ESC line.

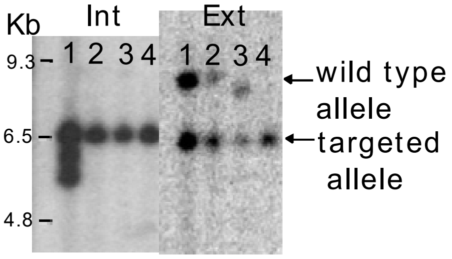

Southern blotting screening of clones from *Aplnr*-tdTomato reporter ESC line production. Genomic DNA of G418-resistant clones was performed using internal (int) and external probe to the Neo sequence. Clones 2, 3 and 4 demonstrated a single integration of the targeting vector into the correct site using the internal probe to the neo gene.

Supplementary Figure S2. Commercial α-APLNR antibody is not specific for APLNR.

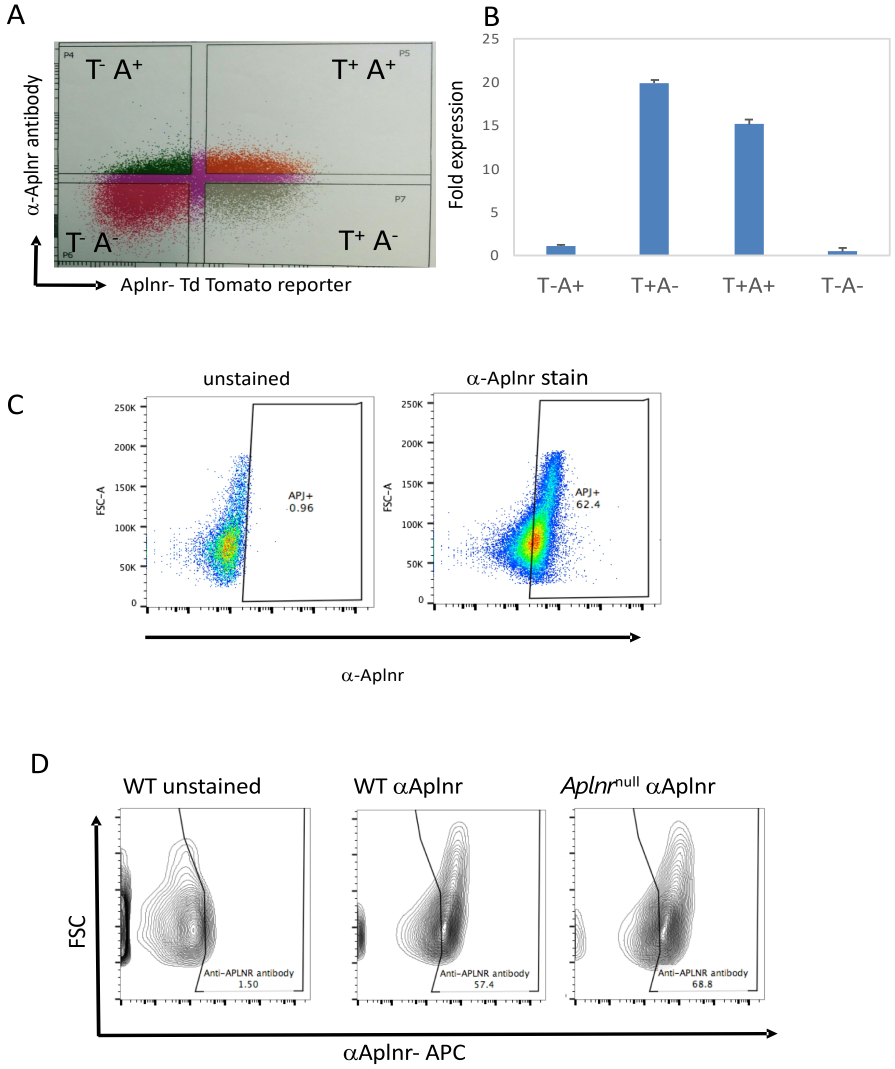

A. Flow cytometry analyses of the *Aplnr*-tdTomato reporter ESC line stained with a commercial antibody to APLNR.

B. qRT-PCR analyses of FAC-sorted cells based on the expression of the tdTomato reporter (T) and α-APLNR antibody staining (A). The presence of *Aplnr* transcripts correlate with the tdTomato expression but not with cells identified by the α-APLNR antibody.

C. Control flow cytometry analyses of 293T cells stained with the α-APLNR antibody demonstrates the poor specificity of the α-APLNR antibody as the 293T cell line does not express *Aplnr* transcripts.

D. Flow cytometry analyses of control, wild type (WT) ESCs and *Aplnr*-null ESCs stained with the anti-APLNR antibody and unstained control.

Supplementary Figure S3. Gating strategy for *Aplnr*-td-Tomato-expressing cells

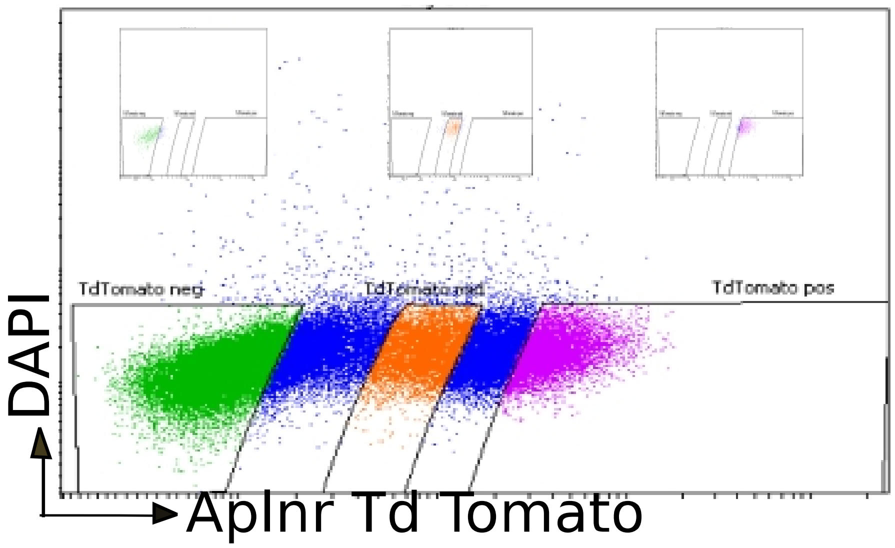

Flow cytometry plot demonstrating the gating for sorting of *Aplnr*-tdTomato high , *Aplnr*-tdTomato intermediate and *Aplnr*-tdTomato negative cell populations. Insert show the re-analysis of sorting cells demonstrating purity of each of the populations.

Supplementary Figure S4. Generation of *Aplnr*-null ESC line.

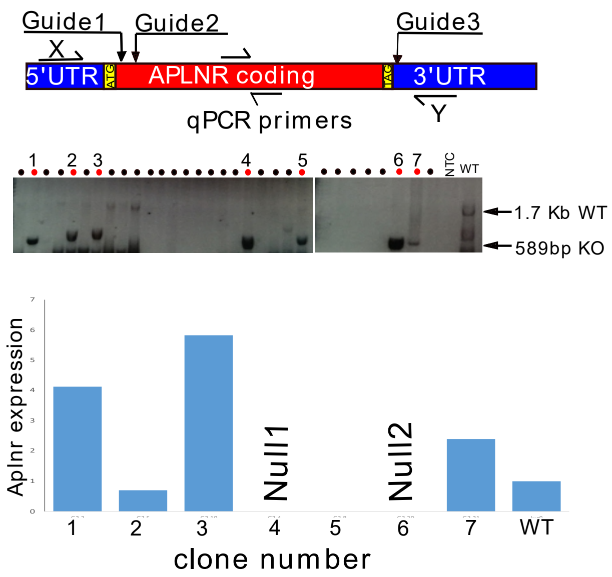

A. Schematic of Crispr/Cas9 strategy to knockout the *Aplnr* coding region showing guide RNAs (Guide 1, Guide 2) and primers that were used to genotype resultant ESC clones and to validate depletion of *Aplnr* transcripts in qRT- PCR experiments.
B. Southern blot showing the presence of the wild type, 1700 base pair restriction fragment in control ESC and the predicted 589 base pair fragment in targeted clones.
C. qRT-PCR of the parental control ESC line (WT) and individual targeted ESC clones (1-7) showing that 2 of these (clones 4 and 6) had no *Aplnr* transcripts, confirming functional homozygosity. These were then defined as Null 1 and Null 2, respectively.

Supplementary Figure S5. Expression of *Aplnr* mRNA transcripts and two potential Apelin and Apela transcripts during mouse ESC differentiation.

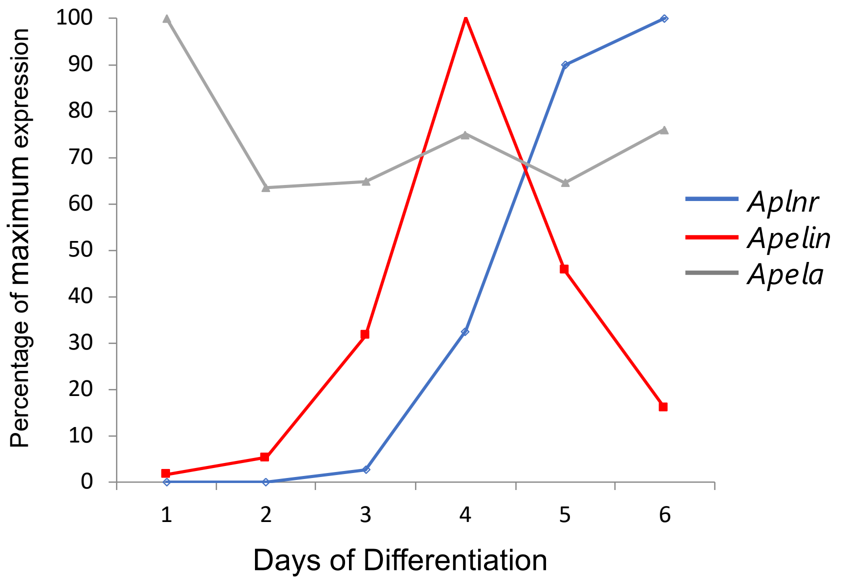

Quantitative RT-PCR analyses of cells undifferentiated mouse ESC (day 0) and at subsequent stages of differentiation in the presence of serum.

Supplementary Figure S6. Addition of APELIN ligands to CFU-C assays increases the number of CFU-M colonies generated from differentiating human PSCs.

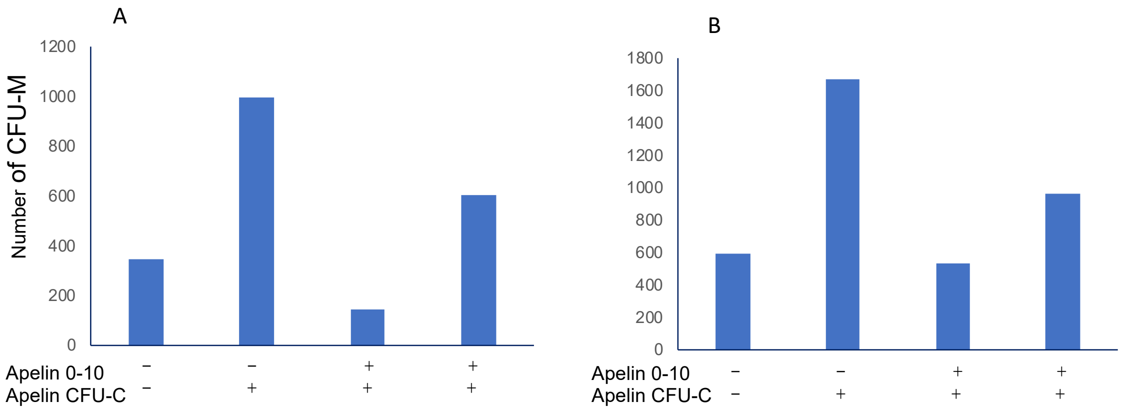

Human H1 ESCs (A) and SFCi55 iPSCs (B) were differentiated for 10 days without APELIN 36 (-) or with the addition of 300 nM APELIN 36 (+) at day 2, 4 and 7 then cells were disaggregated and plated in methylcellulose cultures for a further 10 days in the presence (+) or absence (-) of 300nM APELIN 36 (n=1)
