# Supplementary material for: Modulation of APLNR Signaling Is Required during the Development and Maintenance of the Hematopoietic System
Source: Stem Cell Reports. 2021 Mar 4;16(4):727–40. doi: 10.1016/j.stemcr.2021.02.003 (PMC8072025; doi:10.1016/j.stemcr.2021.02.003)
Supplement: Document S2. Article plus Supplemental information [file mmc2.pdf]

Stem Cell Reports
Article
OPEN ACCESS

Modulation of APLNR Signaling Is Required during the Development and Maintenance of the Hematopoietic System

Melany Jackson,[1,4,5] Antonella Fidanza,[1,5] A. Helen Taylor,[1] Stanislav Rybtsov,[1,2] Richard Axton,[1] Maria Kydonaki,[1] Stephen Meek,[3] Tom Burdon,[3] Alexander Medvinsky,[1,2] and Lesley M. Forrester[1,*]
[1]MRC Centre for Regenerative Medicine, University of Edinburgh, 5 Little France Drive, Edinburgh EH16 4UU, UK
[2]Institute for Stem Cell Research, Centre for Regenerative Medicine, 5 Little France Drive, Edinburgh EH16 4UU, UK
[3]Roslin Institute, University of Edinburgh, Easter Bush, Midlothian EH25 9RG, UK
[4]Present address: Roslin Technologies Limited, Roslin Innovation Centre, University of Edinburgh, Easter Bush, Midlothian EH25 9RG, UK
[5]These authors contributed equally
*Correspondence: l.forrester@ed.ac.uk
https://doi.org/10.1016/j.stemcr.2021.02.003

SUMMARY

Apelin receptor (APLNR/AGTRLl1/APJ) marks a transient cell population during the differentiation of hematopoietic stem and progenitor cells (HSPCs) from pluripotent stem cells, but its function during the production and maintenance of hematopoietic stem cells is not clear. We generated an *Aplnr*-tdTomato reporter mouse embryonic stem cell (mESC) line and showed that HSPCs are generated exclusively from mesodermal cells that express *Aplnr*-tdTomato. HSPC production from mESCs was impaired when *Aplnr* was deleted, implying that this pathway is required for their production. To address the role of APLNR signaling in HSPC maintenance, we added APELIN ligands to *ex vivo* AGM cultures. Activation of the APLNR pathway in this system impaired the generation of long-term reconstituting HSPCs and appeared to drive myeloid differentiation. Our data suggest that the APLNR signaling is required for the generation of cells that give rise to HSCs, but that its subsequent downregulation is required for their maintenance.

INTRODUCTION

Definitive hematopoietic stem and progenitor cells (HSPCs) generate all cells of the blood and immune system, with the most potent of these, the hematopoietic stem cell (HSC), being capable of repopulating the entire blood system upon transplantation. HSPCs arise through a complex process, precisely coordinated at a number of anatomical sites throughout embryonic development. In the mouse embryo the first wave of hematopoietic development originates in the yolk sac around embryonic day 7.25 (E7.25) and gives rise to primitive embryonic erythrocytes, megakaryocytes, and macrophages (Palis, 2014). The second wave also originates from the yolk sac from E8.25 and gives rise to erythro-myeloid progenitors that are defined by their ability to also generate granulocytes and natural killer cells (McGrath et al., 2015; Dege et al., 2020). The third wave originates in the ventral region of the developing dorsal aorta within the aorta-gonad mesonephros (AGM) at E10.5–E11.5, where HSCs, which can repopulate the entire hematopoietic system upon transplantation, emerge (Medvinsky and Dzierzak, 1998; Medvinsky et al., 2008). HSCs arise from hemogenic endothelium through the progressive loss of endothelial markers, such as VE-cadherin and gain of hematopoietic markers, such as CD41 and KIT (Dzierzak and Medvinsky, 2008; Ivanovs et al., 2011; Medvinsky and Dzierzak, 1996; Rybtsov et al., 2011; Taoudi and Medvinsky, 2007). HSCs have been visualized budding from intra-aortic hematopoietic clusters before they are released into the circulation (Bertrand et al., 2010; Boisset et al., 2010; Kissa and Herbomel, 2010; Taoudi et al., 2005, 2008). Research on the molecular mechanisms associated with the development of HSPCs in the embryo has instructed the design of culture protocols to model hematopoiesis *in vitro* from pluripotent stem cells (PSCs) (Ivanovs et al., 2017). However, it has proven challenging to generate bone fide HSCs that are functionally capable of long-term reconstitution. The small number of reports that claim to have succeeded, albeit with very low efficiency, have done so by employing transgenic strategies and/or providing an *in vivo* environment for their maturation (Ditadi et al., 2017; Sugimura et al., 2017; Suzuki et al., 2013). It is unclear whether the failure to detect transplantable HSCs from differentiating iPSCs reflects deficiencies in their generation or whether they are produced but fail to be maintained. Our recent finding that HS-like cells are generated transiently during human iPSC differentiation indicates that their maintenance is a significant problem (Fidanza et al., 2020). Further insight into the cellular and molecular mechanisms associated with both the production and maintenance of HSCs *in vivo* and *in vitro* will aid in designing improved culture conditions for the efficient *in vitro* production of functional HSCs.

The Apelin receptor gene (*Aplnr/Agtrl1/Apj*) encodes a member of the G protein-coupled receptor family and has been implicated in cardiac, endothelial, and hematopoietic development in a number of model systems (D'Aniello et al., 2009; Inui et al., 2006; Quertermous, 2007; Scott et al., 2007). Pertinent to this study is the involvement of Aplnr *Aplnr* in endothelial cell maturation during development and the fact that it appears to be expressed at a higher

This is an open access article under the CC BY license (http://creativecommons.org/licenses/by/4.0/).

level in endothelial cells of the AGM region, implying that it could be related to their hemogenic potential both in mouse (Kidoya et al., 2015; Kidoya et al., 2008, 2010) and human embryos (Crosse et al., 2020). In the adult bone marrow, endothelial cells that express APELIN, one of the APLNR ligands, participate in vascular remodeling after irradiation and drive post-transplant recovery through feedback from HSPCs (Chen et al., 2019). The expression profile of the genes encoding APELIN and its ligands (APELIN and APELA) during early mouse embryonic development indicates that this signaling pathway is also active in the mesoderm and its derivatives (D'Aniello et al., 2009; Devic et al., 1999). In differentiating human PSCs *APLNR* is expressed in Mixl1-expressing mesodermal progenitors, and the addition of APELIN increased the production of blast colonies, which are considered to be derived from a common precursor to endothelial and hematopoietic cells (Yu et al., 2012; Vodyanik et al., 2010). Our previous research has also implicated the APLNR pathway during hematopoietic development *in vitro*. We showed that the expression of genes encoding APLNR and one of its ligands, APELIN, correlated with the increased production of HSPCs when the transcription factor, HOXB4 was activated in differentiating mouse and human PSCs (Jackson et al., 2012, 2016). Although all of these studies implicate a role for APLNR signaling during hematopoietic development, the specific function of this pathway has not been addressed directly. To this end, we generated an *Apln*r-tdTomato reporter mouse ESC (mESC) line and have shown that the *Aplnr*-tdTomato reporter marks a population of differentiating mesoderm cells that has the potential to form hematopoietic and endothelial lineages. We then generated an *Aplnr*-null mESC line and demonstrated that HSPCs production was significantly impaired, implying that this signaling pathway is indeed required for their generation. To assess the role of APLNR signaling in HSC maturation and maintenance, we added APELIN ligands to AGM explant cultures and observed a marked decrease in the number of long-term reconstituting HSCs and an increase in the differentiation of myeloid cells.

RESULTS

Aplnr-tdTomato Reporter Marks Mesodermal Cells in Differentiating ESCs

To define the phenotype of cells expressing the APLNR receptor during hematopoietic differentiation of ESCs we generated an *Aplnr*-tdTomato reporter mESC line using CRISPR-Cas9-mediated genome editing (Figure 1A). mESCs were transfected with the *Aplnr* targeting vector, Cas9 plasmid, and specific gRNAs. Thirty-five G418-resistant colonies were selected and 18 of these were screened by genomic PCR and Southern blotting (Figure S1A). We confirmed that the *Aplnr*-tdTomato reporter faithfully mimicked *Aplnr* transcript expression by demonstrating the expression of *Aplnr* transcripts in fluorescence-activated cell sorting (FACS)-sorted tdTomato-positive, but not tdTomato-negative cells (Figure 1B). However, we noted that the expression of the *Aplnr*-tdTomato reporter did not correlate with the expression of a protein detected by a commercially available α-APLNR antibody (Figure S2). Real-time PCR analyses of FACS-sorted cells detected *Aplnr* transcripts in cells sorted based on expression of the *Aplnr*-tdTomato reporter but not α-APLNR antibody staining, suggesting that the α-APLNR antibody was binding non-specifically to the cell surface (Figures S2A and S2B). This was supported by demonstrating that the α-APLNR antibody detected a protein in 293T cells that do not express *Aplnr* transcripts (Figure S2C). We were finally convinced that this commercial antibody was not specific for APLNR when we demonstrated that it detected a protein in differentiating *Aplnr*-null ESCs (see below) (Figure S2D). These findings emphasized the requirement of the *Aplr*-tdTomato reporter mESC lines in defining the potential of APLNR-expressing cells.

The timing of expression of the *Aplnr*-tdTomato reporter during mESC differentiation was compared with the cell surface expression of E-cadherin (ECAD), an epithelial marker that is expressed at a high level in undifferentiated PSCs but downregulated upon differentiation into mesoderm lineages (Malaguti et al., 2013). Because the APLNR is first expressed in the developing mesoderm of the mouse embryo, we predict a mutually exclusive expression pattern with ECAD. As expected, we observed ECAD expression in undifferentiated ESCs (day 0) and expression was gradually reduced as differentiation progressed (Figure 1C). In contrast, the *Aplnr*-tdTomato reporter was not expressed in undifferentiated cells, and expression increased during differentiation. *Aplnr*-tdTomato-expressing cells were first detected at day 3 of differentiation in both $ECAD^{+}$ and $ECAD^{-}$ cells, but by day 5 virtually all the *Aplnr*-tdTomato-expressing cells were $ECAD^{-}$ (Figure 1C). Thus, differentiating ESCs transition from $ECAD^{+}APLNR^{-}$ to $ECAD^{+}APLNR^{+}$ phenotype, which then downregulate ECAD to become $ECAD^{-}APLNR^{+}$. The highest proportion (20%) of the intermediate, double-positive cells was present around day 3.

To confirm that the *Aplnr*-tdTomato reporter is expressed in the emerging multipotent mesoderm, we show that most of the cells expressing a high level of *Aplnr*-tdTomato at day 3 co-expressed high levels of FLK1 and that around half of these also express PDGFRα (Figure 1D). As differentiation progressed, *Aplnr*-tdTomato was observed in cells expressing the endothelial cell markers (VE-cadherin and FLK1), with the majority of $VE\text{-}CAD^{+}$ and $FLK1^{+}$ cells expressing the reporter at day 4 (Figure 1E). Hematopoietic cells expressing CD41 were first detected in this differentiation protocol at day 6 and the *Aplnr*-tdTomato reporter

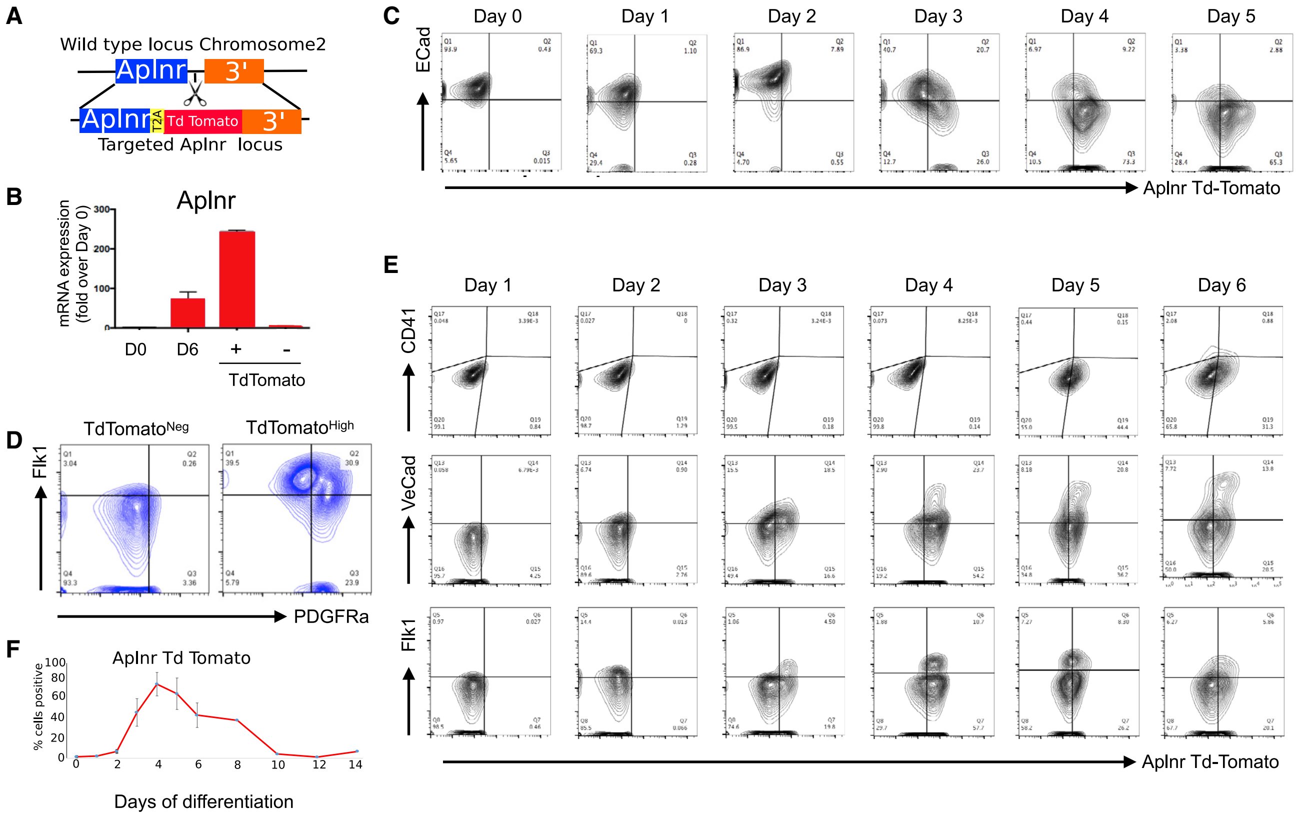

Figure 1. *Aplnr*-tdTomato Reporter ESC Line Mimics the Activity of the Aplnr Locus during ESC Differentiation and Tracks a Transient Mesoderm Cell Population

(A) CRISPR-Cas9 gene-editing strategy. Guide RNAs were designed to cut the genomic region of chromosome 2 immediately after the *Aplnr* coding sequence. Schematic of targeting vector that was used to insert the tdTomato reporter gene at this site, followed by a T2A sequence and Neo^{R} cassette (not shown).

(B) Quantitative RT-PCR of *Aplnr* expression in undifferentiated Aplnr-tdTomato reporter ESCs (day 0 [D0]), unsorted differentiated day 6 (D6) cells and day 6 cells sorted into Aplnr-tdTomato-positive (+) and -negative (−) populations (n = 3). Error bars represent standard deviation of data from three independent experiments.

(C) Representative flow cytometry plots of *Aplnr*-tdTomato and E-cadherin expression during differentiation from undifferentiated ESCs (D0) to day 5 of differentiation.

(D) Flow cytometry of *Aplnr*-tdTomato ESCs at day 3 of differentiation stained with antibodies to FLK1 and PDGFRα and then analyzed after gating on either APLNR negative or APLNR high.

(E) Representative flow cytometry plots of *Aplnr*-tdTomato together with CD41, VE-cadherin, or FLK1 during differentiation from days 1 to 6 of differentiation.

(F) Proportion of *Aplnr*-tdTomato-positive cells detected by flow cytometry in undifferentiated (D0) cells and during a 14 day time course of differentiation. Error bars represent standard deviation of data from three independent experiments.

was detected in a proportion of these cells (Figure 1E). Overall, the highest proportion (around 70%) of *Aplnr*-tdTomato cells was observed at day 4 and this gradually declined as differentiation proceeded (Figure 1F).

Hematopoietic Activity Is within the Cell Populations that Express High Levels of the *Aplnr*-td-Tomato Reporter

Aplnr-tdTomato mESCs were differentiated into embryoid bodies (EBs) for 5 days, dissociated, and cells expressing different levels of *Aplnr*-tdTomato were isolated by flow cytometry (Figure S3). The differentiation potential of FACS-sorted cells was assessed using CFU-C assays (Figure 2A). Hematopoietic activity was found almost exclusively within in the *Aplnr*-tdTomato-expressing cell population with virtually no CFU-Cs generated from the sorted *Aplnr*-tdTomato-negative cell population. There was a significantly higher overall number of CFU-Cs generated from cells expressing a high level of *Aplnr*-tdTomato compared with cells expressing low levels or that were negative for Aplnr-

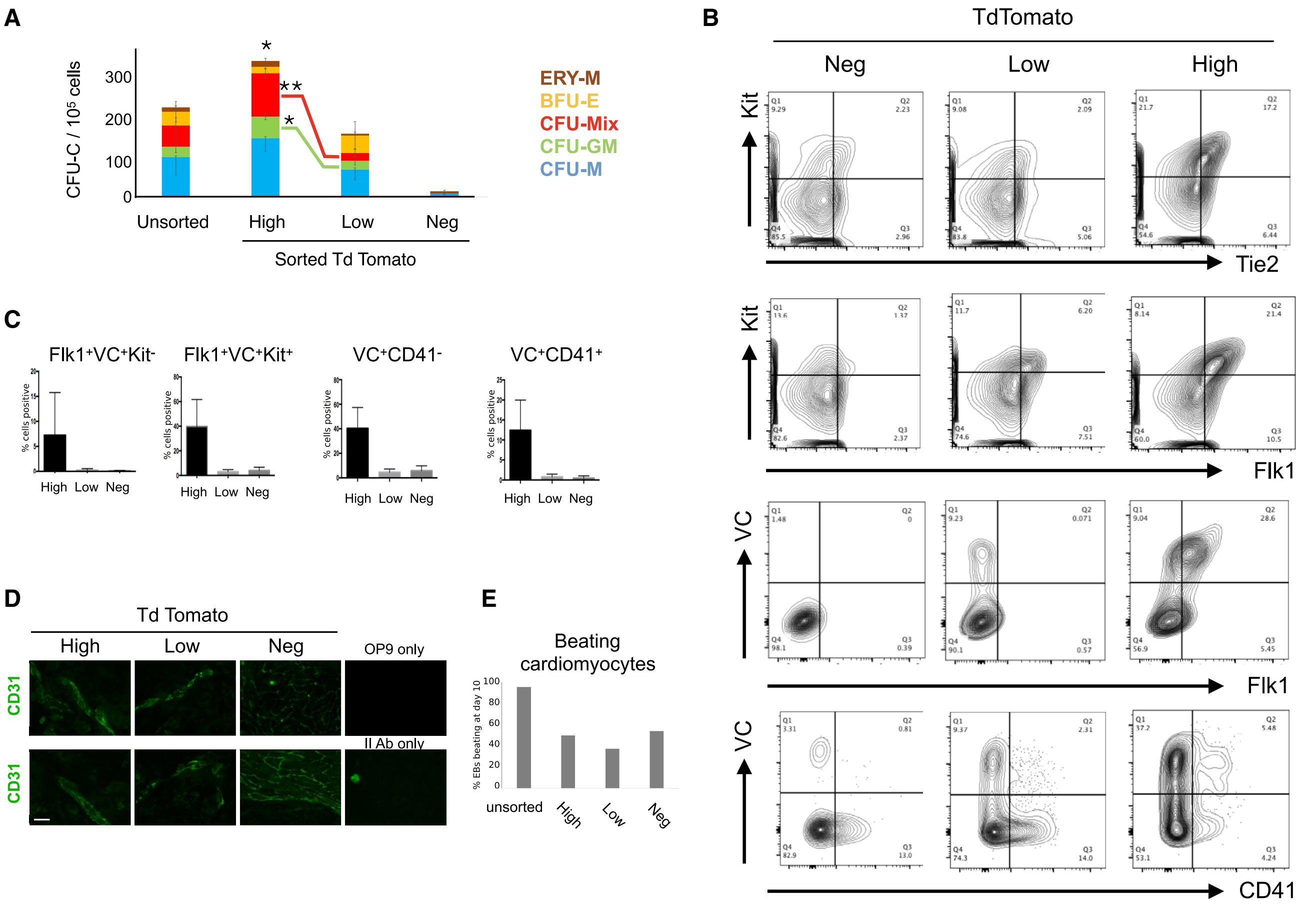

Figure 2. *Aplnr*-tdTomato Marks Mesoderm Fated to Become Hemogenic Endothelium

(A) Number of CFU-Cs generated from 10^5 unsorted differentiating ESCs (day 6) and cell populations sorted biased a high (Hi), low (Lo), or negligible (Neg) level of tdTomato expression (n = 3; *p < 0.05). Statistically significant difference in number of CFU-Mix (**p = 0.003) and CFU-GM (*p < 0.03) colonies from *Aplnr*-tdTomato high and low cells. No difference was observed in the number of uni-lineage BFU-E and CFU-M (see Figure S3 for FACS sorting strategy).

(B) Representative flow cytometry plots of differentiating Aplnr-tdTomato ESCs (day 6) co-stained with antibodies against KIT, TIE2, VE-CAD, FLK1, and CD41. Backgating of cells expressing high, low, or negligible (Neg) levels of Aplnr-tdTomato demonstrates that cells expressing TIE2, FLK1, VE-CAD (VC), KIT, and CD41 are primarily found in the *Aplnr*-tdTomato-high cell population.

(C) Quantification of flow cytometry data in (B) showing the percentage of cell type within each of the subpopulations defined by the level of *Aplnr*-tdTomato expression (n = 3; *p < 0.05).

(D) Immunohistochemistry using an αCD31 antibody of differentiating ESCs after FACS and culturing on OP9 stromal cells in the presence of VEGF, demonstrating the endothelial potential differentiating cells expressing high and low levels of *Aplnr*-tdTomato. Two replicate experiments are shown. Scale bar represents 100 μm (×40 magnification).

(E) Percentage of embryoid bodies (EBs) with associated beating cardiomyocytes in EBs generated from unsorted day 6 differentiating ESCs or cells at this stage that were sorted based on the expression of high (Hi), low (Lo), or negligible (Neg) levels of *Aplnr*-tdTomato expression.

tdTomato. The number of multilineage (CFU-Mix) colonies and CFU-GM colonies was significantly higher from cells expressing a high level of *Aplnr*-tdTomato compared with the cells expressing a low level of *Aplnr*-tdTomato, whereas there was no significant difference in the production of single lineage colonies (Figure 2A).

Backgating of cells expressing high, low, or negligible (Neg) levels of *Aplnr*-tdTomato demonstrated that cells expressing TIE2, FLK1, VE-CAD (VC), KIT, and CD41 are primarily found in the *Aplnr*-tdTomato-high cell population (Figure 2B). A subset of these cells also expressed the hematopoietic markers CD41 and KIT, implying that APLNR is expressed at the transitional state from an endothelial to hematopoietic phenotype (Figures 2B and 2C).

Aplnr-tdTomato-expressing cells were plated onto irradiated OP9 cells, cultured in the presence of VEGF for

10 days, and then immuno-stained for the endothelial marker, CD31, to assess their potential to form endothelial cells (Figure 2D). Cells expressing high and low levels of *Aplnr*-tdTomato generated endothelial structures consisting of $CD31^+$ cells but no such structures were produced by *Aplnr*-tdTomato-negative cells. To assess whether the *Aplnr*-tdTomato reporter was marking all mesoderm progenitors or a subpopulation committed to hematopoietic and endothelial lineages, we assessed the potential of sorted cells to differentiated into another mesodermal cell type. EBs were dissociated at day 6, sorted based on the expression of *Aplnr*-tdTomato, and then assessed for their potential to generate cells of the cardiac lineage. Although sorting reduced the overall production of beating cardiomyocytes compared with unsorted cells, there was no correlation between the level of *Aplnr*-tdTomato expression and the potential to generate cardiac cells suggesting that cardiomyocytes could be generated from mesoderm that did not express *Aplnr*-tdTomato (Figure 2E). Taken together, these data support our hypothesis that the *Aplnr*-tdTomato reporter marks mesoderm and that *Aplnr* expression is specifically required for the efficient endothelial and hematopoietic differentiation.

Aplnr Is Required for Hematopoietic Cell Production from ESCs

To confirm that APLNR signaling is required during the differentiation of hematopoietic cells from PSCs, we generated *Aplnr*-null mESCs using a CRISPR-Cas9 strategy. *Aplnr* is a single-exon transcript with a coding region of 1,131 base pairs (bp), so our strategy involved excising the complete coding region using guide RNAs directed to the 5′ and 3′ ends (Figure S4A). pSPCas9-2A-mCherry-U6-gRNA plasmids containing pre-selected gRNAs (see the Experimental Procedures) were transfected into E14 mESCs. Cells that had been successfully transfected were sorted based on mCherry expression 24–48 h after transfection and then plated at low density. Individual mESC clones were isolated and genomic DNA was screened by PCR using primers that spanned the deleted region. The PCR assay was designed to amplify a 589 bp product from the deleted, knockout (KO) allele and a 1,700 bp amplicon from the wild-type (WT) allele. The KO allele was identified in 7 out of 30 mCherry-positive clones (Figure S4B). This assay did not distinguish between heterozygous and homozygous clones because the smaller 589 bp amplicon associated with the KO allele would likely be amplified preferentially over the larger WT allele. To differentiate between functionally heterozygous and homozygous mESC clones, we sequenced the amplicons and found that, in the majority of clones that had one deleted *Aplnr* allele, the second *Aplnr* allele had an insertion or a deletion that resulted in a frameshift mutation (data not shown). Quantitative real-time PCR was used to select clones in which the targeting events resulted in ablation of *Aplnr* transcripts and could therefore be considered as functionally null at the *Aplnr* locus (Figure S4C).

Aplnr-null mESCs could be maintained as undifferentiated ESCs in the presence of leukemia inhibitory factor (LIF) in a comparable manner with controls (data not shown). However, when induced to differentiate and assessed in hematopoietic colony assays, significantly lower numbers of hematopoietic CFU-C colonies (CFU-M, CFU-GM, and CFU-Mix) were formed from two independently derived *Aplnr*-null ESCs compared with control WT ESCs at day 6 of differentiation (Figure 3A). Flow cytometry analyses confirmed that the *Aplnr*-null ESCs produced a lower proportion of $CD41^+$VE-$cadherin^+$ hematopoietic progenitor cells (HPCs) (Figures 3B and 3C).

When subjected to a macrophage-specific differentiation protocol, the proportion of mature $F4/80^+CD11B^+CD16^+$ macrophages generated from *Aplnr*-null ESCs was significantly lower than controls (Figure 3D). To confirm that the reduced hematopoietic differentiation was due to the absence of *Aplnr* and not simply a consequence of ESC clonal variation, we introduced an APLNR-expressing plasmid into the *Aplnr*-null ESC clones and demonstrated that the production of $F4/80^+CD11b^+$ macrophages was increased in the presence of the exogenous *Aplnr* (Figure 3D). These data indicate that the APLNR pathway is required for the development of HPCs and for the differentiation of the myeloid lineage.

Activation of the APLNR Pathway Has No Significant Effect on the Production of Hematopoietic Progenitors in Differentiating Mouse and Human PSCs

As HSPC production is impaired when *Aplnr* is deleted, we hypothesized that their production might be increased when the APLNR pathway is activated by known APLNR ligands, such as APELIN. However, when APELIN was added to the serum-free cytokine-based hematopoietic differentiation of mESCs, we observed no effect on the number or phenotype of CFU-Cs (Figure 4A). This was somewhat unexpected given that previous studies had reported promotion of the production of blast colonies when Apelin was added to differentiating human ESCs (Yu et al., 2012). To confirm that the effects we had observed were not species specific we replicated our findings using both human ESCs and induced pluripotent stem cells (iPSCs), again demonstrating that the addition of APELIN peptides had no effect on CFU-C formation (Figure 4B). This apparent discrepancy might be partly explained by the fact that two of the proposed ligands (APELIN and APELA) for APLNR are expressed in differentiating ESCs (Figure S5). This could suggest that the APLNR signaling pathway is already activated and that any further addition of ligand

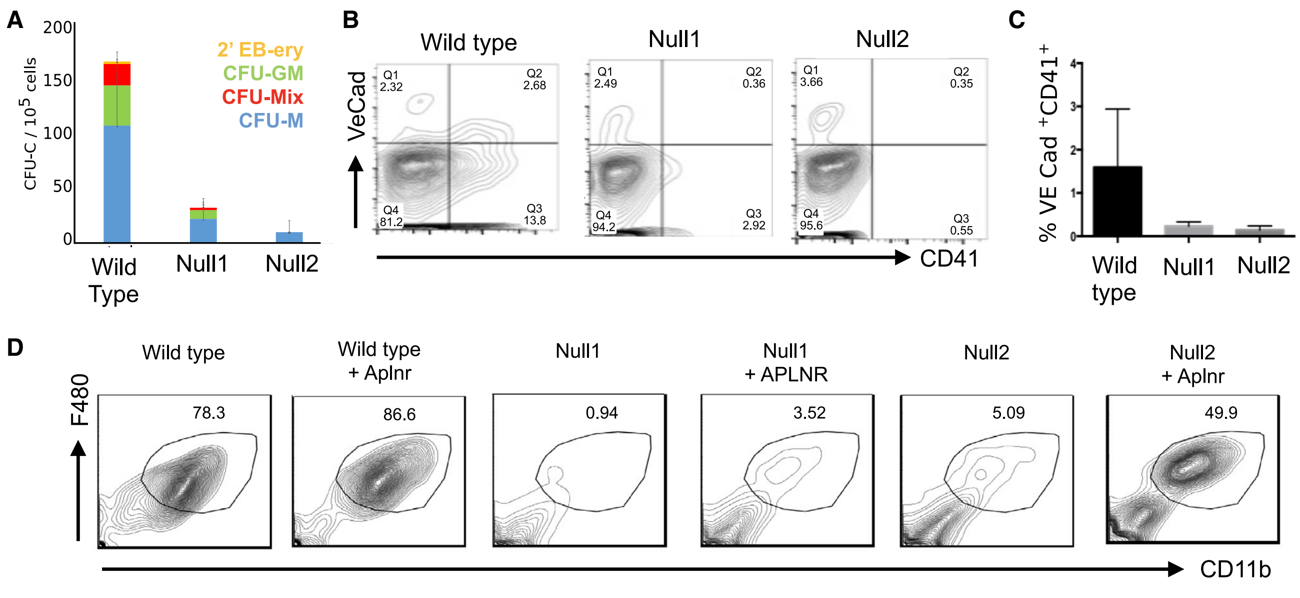

Figure 3. Targeted Deletion of *Aplnr* Results in Impaired Hematopoietic Differentiation

(A) Number of hematopoietic CFU-C colonies generated from 10^5 differentiated (day 6) control ESCs and two independently derived *Aplnr*-null (Null1 and Null2) ESC clones. CFU-M (macrophage), CFU-GM (granulocye/macrophage), CFU-GEMM (multilineage, including granulocyte, erythroid, macrophage, and megakaryocytes), 2′ EB-ery (secondary embryoid bodies with associated erythroid cells) (*$p < 0.05$). Error bars represent standard deviation of data from four independent experiments.

(B) Representative flow cytometry analyses of day 6 differentiated control ESCs and two independently derived *Aplnr*-null ESC clones (Null1 and Null2) using antibodies to VE-CAD and CD41.

(C) Quantification of flow cytometry analyses (B) showing the percentage of VE-CAD$^+$CD41$^+$ cells at day 6 of differentiation from control and two *Aplnr*-null ESC clones (*$p < 0.05$). Error bars represent standard deviation of data from three independent experiments.

(D) Representative flow cytometry of cells generated following the macrophage differentiation protocol of control (wild type), two *Aplnr*-null ESC lines (Null1 and Null2), and the Null1 and Null2 ESCs that were transfected with an APLNR-expressing plasmid (+APLNR).

fails to exert an observable functional effect. Interestingly, when APELIN ligands were included in the CFU-C assay, an increase in macrophage colonies was observed, implying that activation of the pathway might drive the differentiation of HPCs into myeloid lineages (Figure S6).

Genes expressing APLNR and Its Ligands Are Expressed at the Site of HSC Emergence *In Vivo*

To assess the role of the APLNR pathway in HSPC maintenance and differentiation, we turned to the mouse AGM explant system that is known to support both the maturation and maintenance of definitive HSPCs (Taoudi et al., 2008). We first demonstrated that the genes encoding APLNR and its ligands, *APELIN* and *APELA*, were expressed in the region of the developing embryo at E9.5 and E11.5 when definitive HSCs first emerge (Figure 5A). Low, but detectable levels of expression were also observed in the yolk sac and fetal liver. These data support the hypothesis that the APLNR signaling pathway is active during the emergence of HSCs *in vivo*. To further define which cell types within the AGM express the genes encoding APLNR and its ligands we analyzed published datasets from single-cell RNA sequencing of mouse AGM tissue (GEO: GSE143637) (Vink et al., 2020), human AGM tissue (GEO: GSE151877; sample GSM4592621) (Crosse et al., 2020) and differentiating human iPSCs (Database: EMBL EBI Arrayexpress; E-MTAB-9295 (Fidanza et al., 2020). *Aplnr/APLNR* is expressed at a relatively high level in most of the cells associated with endothelial cell clusters in both mouse and human AGM and in differentiating human iPSCs (Figures 5B–5D). RNA encoding the ligand APELIN was expressed in more restricted subpopulations of endothelial cells in all datasets, whereas RNA encoding APELA was barely detectable (Figures 5B–5D). Interestingly, within HSPC clusters, RNA encoding APLNR and APELIN (but not APELA) was detected in mouse but not human AGM tissue. *APLNR* transcripts were detected in some iPSC-derived HSPCs, supporting the idea that *APLNR* could be expressed in cells at the EHT transition.

Activation of the APLNR Pathway Reduces the Production of Transplantable HSCs in Aggregate Cultures

To assess whether activation of the APLNR pathway could influence the production, maintenance, or differentiation

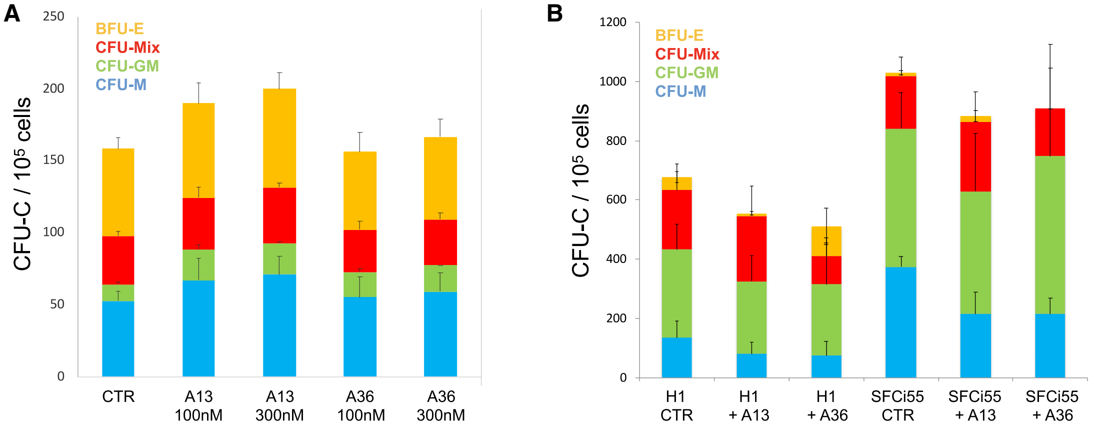

Figure 4. Addition of APELIN Ligands to Differentiating Mouse and Human PSCs Does Not Enhance Hematopoietic Colony Production

(A) Number of CFU-Cs generated in differentiating mESCs with no APELIN ligands added (control) and after the addition of APELIN 13 (A13) or APELIN 36 (A36) at 30 and 100 nM (n = 3).

(B) Number of CFU-Cs generated in differentiating human ESCs (H1) (n = 8) and human iPSCs (SFCi55) (n = 3) in control cultures with no APELIN ligands added (no A) and after the addition of APELIN 13 (A13) or APELIN 36 (A36). Error bars represent standard deviation of data from three independent experiments (A and B).

HSCs, we added APLNR ligands, APELIN 13 (A13), APELIN 36 (A36), or APELA 21 (A21), which are known to support *ex vivo* maturation and expansion of definitive HSCs, to AGM reaggregation cultures (Figure 6A) (Rybtsov et al., 2014; Taoudi et al., 2008). The caudal region of E9.5 embryos that includes AGM tissue was dissected and placed in the aggregation culture in the presence of cytokines, with or without Apelin peptides, for 7 days. Resultant cells were assessed for CFU-C formation, flow cytometry, and their ability to reconstitute lethally irradiated recipients *in vivo*. The addition of either A13 or A36 peptides to the aggregate cultures resulted in a slight, but not significant, increase in the total number of hematopoietic progenitors as assessed by CFU-C production (Figure 6B). Flow cytometry analyses of cells derived from the CFU-C colonies demonstrated an increase in the proportion of mature myeloid cells expressing high levels of CD11b, a proportion of which also expressed Gr1 (Figures 6C and 6D). The increase in the production of mature myeloid cells in the presence of APLNR ligands coincided with a decrease in the production of immature ($LIN^-/SCA^+/KIT^+$) HPCs (Figure 6E).

To assess the effects of APLNR ligands on the production of functional reconstituting HSCs in *ex vivo* cultures, day 9.5 AGM cells (CD45.2) were cultured for 7 days in the presence of various concentrations of A13, A36, or A21, and then resultant cells were transplanted into irradiated CD45.1/2 recipients. Almost all (8/9) recipients that received control cell aggregates cultured without Apelin peptides demonstrated successful HSC reconstitution at 14 weeks after transplantation (>10% $CD45^+$). In contrast, when a high concentration (300 nM) of A13, A36, or A21 was added to the aggregate culture, the number of successfully transplanted animals was reduced to 3/6, 1/10, and 2/6, respectively (Figure 5G). The effect of peptide addition was dose dependent, with the addition of a lower concentration (100 nM) of all peptides having a less profound detrimental effect on the ability of cultured cells to reconstitute the hematopoietic system of irradiated recipient mice (Figure 5G). To define the timing of this detrimental effect, we isolated pre-HSC type I ($VC^+CD41^{lo}CD43^+CD45^-$) and pre-HSC type II cells ($VC^+CD41^{lo}CD43^+CD45^+$) from day 11.5 AGM tissue and assessed the effect of A36 on their subsequent maturation into functional HSCs within aggregate cultures (Figure 5H). In control cultures, pre-HSC type I could be matured *ex vivo* into reconstituting HSCs, but in the presence of APELIN 36 resultant cells did not reconstitute irradiated mice. A comparable, but less profound effect of A36 was observed on pre-HSC type II when again reconstitution was significantly reduced. These data demonstrate that activation of the APLNR pathway by the addition of ligands is detrimental to the generation and maintenance of functional HSCs in *ex vivo* cultures.

Taken together, the results of our study indicate that the APLNR pathway is required for the production of hematopoietic cells but that the activation of the APLNR pathway at a later stage negatively affects HSC function. The increased production of myeloid cells in the presence of APLNR ligands both in explant cultures and in differentiating human ESCs suggests that this

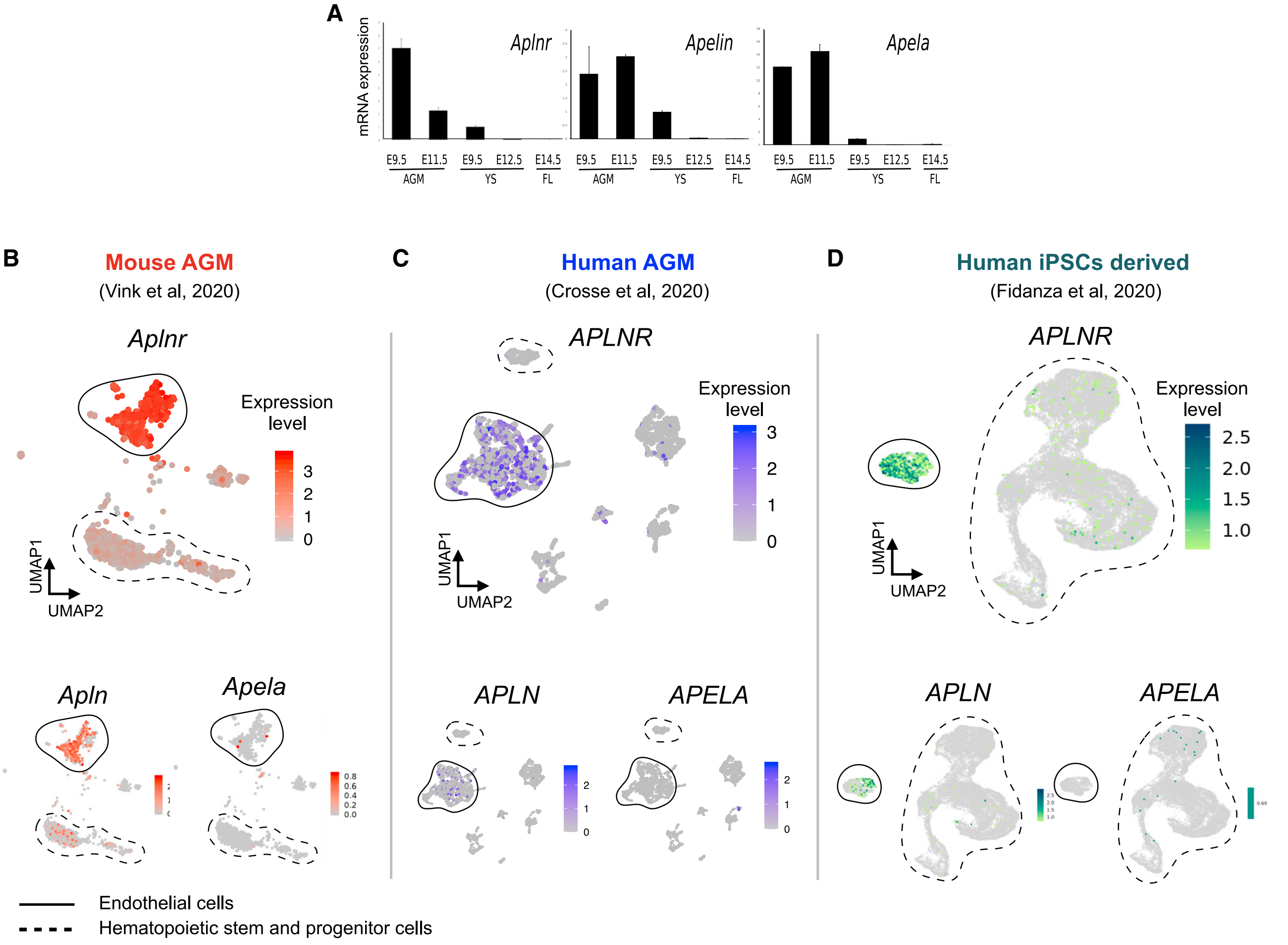

Figure 5. Expression of Aplnr, Apelin, and Apela in Hematopoietic Tissue

(A) Quantitative RT-PCR analyses of RNA isolated from the AGM region, yolk sac (YS), and fetal liver (FL) dissected from embryos at the indicated stage of development using primers to detect transcripts encoding APLNR and its ligands, APELIN and APELA (n = 2).

(B–D) UMAP plots of single-cell sequencing data derived from mouse (B) and human (C) AGM tissue and differentiating human iPSCs (D) demonstrating *Aplnr/APLNR*, *Apelin/APELIN*, and *Apela/APELA* expression in endothelial cell clusters. Endothelial cell and HSPC clusters are highlighted with solid and dotted outlines, respectively.

signaling pathway favors differentiation over self-renewal.

DISCUSSION

We previously identified the genes encoding APLNR and its ligand APELIN as the most highly upregulated genes when HOXB4 was activated in differentiating mESCs (Jackson et al., 2012). As this increase in expression correlated with an enhanced hematopoietic differentiation, we hypothesized that the APLNR signaling pathway could be associated with the production and/or maintenance of HSPCs. Here, we show that the *Aplnr*-tdTomato reporter marks differentiating mesodermal cells that have the potential to differentiate into hematopoietic and endothelial lineages and that deletion of the *Aplnr* gene impairs the *in vitro* production of HSPCs from mESCs. Our findings are consistent with previous studies that have reported a role for this signaling pathway in mesoderm progenitors in both mouse and human differentiating ESCs (Yu et al., 2012; Vodyanik et al., 2010; D'Aniello et al., 2013).

We show that the cells expressing the highest level of the *Aplnr*-tdTomato reporter also express the highest level of endothelial markers. Our analyses of single-cell sequence data from mouse and human AGM tissue and differentiating iPSCs further confirmed *Aplnr/APLNR* expression in endothelial cells. We noted that a proportion of *Aplnr*-tdTomato-positive cells also expressed hematopoietic markers

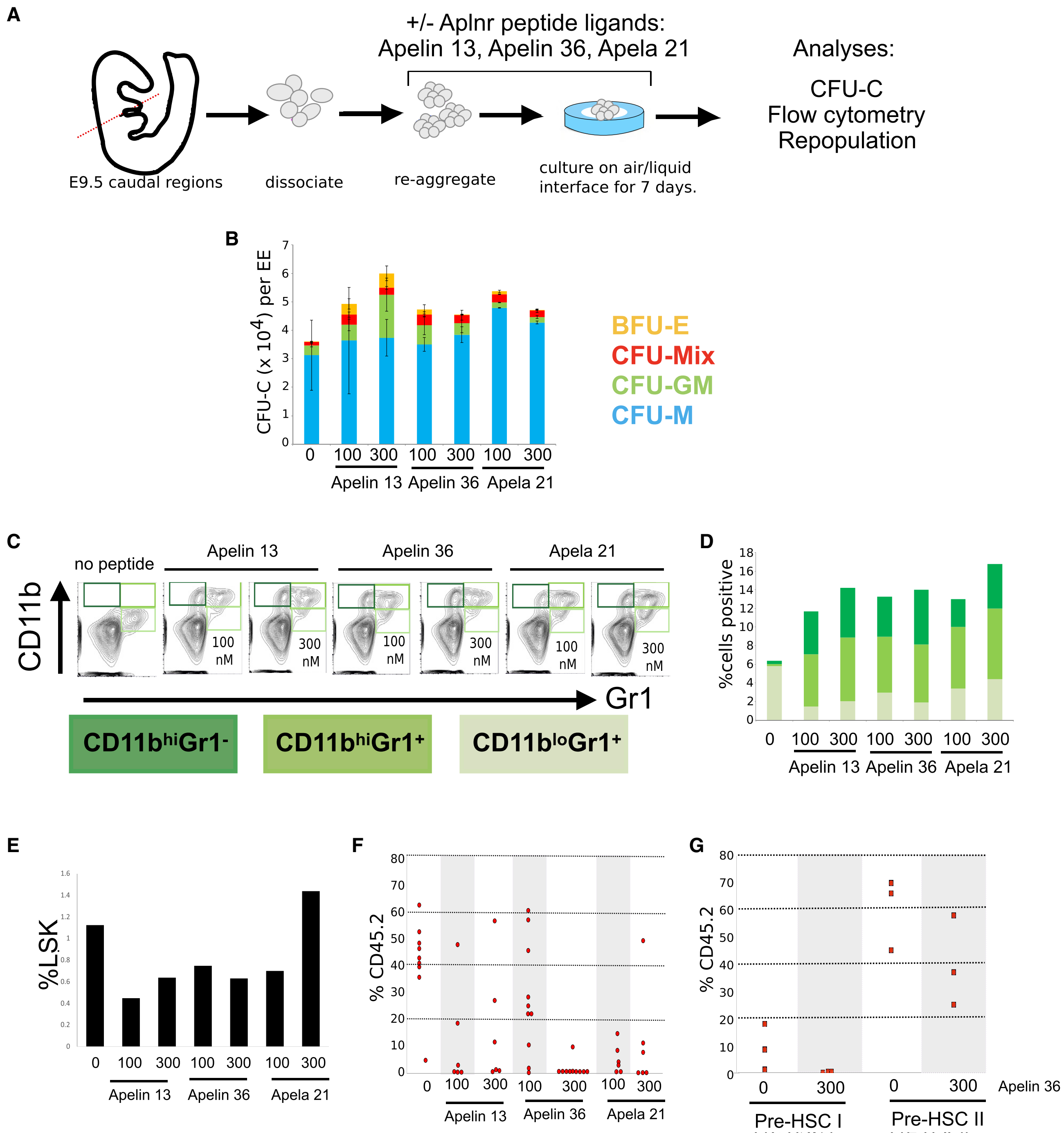

Figure 6. Addition of Aplnr Ligands to AGM Reaggregate Explant Cultures Results in a Reduction in Transplantable HSCs

(A) Schematic of experimental strategy for E9.5 dissection (as example) where the caudal region of the embryo was dissected, tissue dissociated, set up in reaggregate cultures in the presence of Apelin peptides for 7 days and then assayed by for colony formation (CFU-C), flow cytometry, and transplantation into lethally irradiated mice.

(B) CFU-C analyses of reaggregate cultures in the absence (0) or presence (100 or 300 nM) of APELIN 13, APELIN 36, or APELA 21. Error bars represent standard deviation of data from three independent experiments.

(C) Representative flow cytometry plots of live cells obtained after 7 days of E9.5 aggregate culture in presence of APELIN 13, APELIN 36, or APELA 21 using antibodies against the myeloid markers, CD11B and GR1).

(D) Quantification of flow cytometry in (C).

(legend continued on next page)

implying that it is expressed at the EHT transition stage. This is in keeping with single-cell sequencing analyses of human AGM implying that *APLNR* was expressed in hemogenic endothelium (Crosse et al., 2020). Two further studies have reported the downregulation of *Aplnr* as endothelial cells acquire hematopoietic markers during EHT (Bruveris et al., 2020; Oatley et al., 2020). The slight difference in the timing of *Aplnr* downregulation in cells that have acquired hematopoietic markers that we report might reflect the slightly longer half-life of tdTomato compared with *Aplnr* transcripts.

We observed no change in the numbers or phenotype of CFU-C formation after the addition of APELIN ligands to mouse or human ESCs and iPSCs, whereas a previous study reported that Apelin promoted hematopoietic differentiation of human ESCs (Yu et al., 2012). We believe that this apparent discrepancy actually highlights the importance APLNR signaling at the transitional state between endothelial and hematopoietic fate because the positive effect of APELIN on hematopoietic differentiation of human ESCs was reflected in an increase in the number of bipotential blast colonies (Yu et al., 2012). We have shown that transcripts encoding APELIN ligands are present within differentiating mouse and human ESC/iPSC cultures, and it is possible that this would mask the effects of exogenous ligand addition.

In AGM explant reaggregation cultures we show that addition of APLNR ligands results in a reduction in HSCs capable of long-term reconstitution and a concomitant increase in the production of more mature myeloid cells. This finding suggests that activation of the APLNR pathway is detrimental to the maintenance of HSCs and provides the first indication that the precise modulation of APLNR signaling is critical in steady-state hematopoiesis. The increase in the production of myeloid cells by APELIN ligands in *ex vivo* cultures is consistent with our observation that the *in vitro* production of macrophages from ESCs is significantly impaired in the absence of APLNR and that the addition of APELIN to the CFU-C assay resulted in an increase in macrophage colony formation. In future it would be interesting to assess whether the addition of APLNR ligands affects cell cycle, the speed of which has been shown to be important for the emergence of functional definitive HSCs within the hematopoietic clusters the AGM region (Batsivari et al., 2017).

The differential requirements for APLNR signaling at precise stages of HSC emergence and maintenance is comparable with that reported for other signaling pathways, such as the NOTCH signaling pathway, where stage-specific effects are observed in the production of adult HSCs *in vivo* (Souilhol et al., 2016) and in HSPC production *in vitro* from ESCs (Huang et al., 2013).

Critical to the interpretation and the relevance of our findings to hematopoietic development and maintenance *in vivo* is the anatomical and temporal expression of APLNR ligands. Pertinent to this point is the finding, using *Apln-CreER Rosa26*-mTmG reporter mice, that APELIN is expressed in a subpopulation of endothelial cells in adult bone. Conditional knockout of this endothelial cell population revealed their critical role in hematopoietic regeneration following myeloablation (Chen et al., 2019). It is not clear which of the ligands are important in the activation of APLNR in different cell types and it is not known which of the many downstream intracellular pathways are responsible for exerting the ultimate biological effects. A conditional knockout approach for the APLNR receptor and the production of mice carrying the *Aplnr*-tdTomato reporter will be instrumental in gaining a fuller understanding the complexity of this pathway during hematopoietic development and maintenance *in vivo*.

EXPERIMENTAL PROCEDURES

mESC Maintenance and Differentiation

The mESC line E14IV was maintained in Glasgow Minimum Essential Medium (Gibco) supplemented with 10% fetal calf serum (FCS) (Lonza), 2 mM sodium pyruvate (Gibco), 4 mM L-glutamine (Gibco), 1% non-essential amino acids (Gibco), and 0.1 mM β-mercaptoethanol (Sigma) and 100 U/mL of LIF, as described previously (Jackson et al., 2012).

Hematopoietic Differentiation of mESCs

Two days before differentiation, mESCs were plated in N2B27 medium supplemented with 100 U/mL LIF and 10 ng/mL BMP4 to allow cells to adjust to serum-free conditions. To initiate differentiation, at day 0 cells were disaggregated with Accutase (Gibco) then resuspended in N2B27 containing 2.5 ng/mL Activin A, 10 ng/mL basic-helix-loop-helix (bFGF), and 5 ng/mL BMP4 and plated in ultra-low attachment plates (STEMCELL Technologies). Two days later, EBs were plated on gelatinized tissue culture plates in N2B27 containing 10 ng/mL bFGF, 5 ng/mL BMP4, and 15 ng/

(E) Proportion of $LIN^{-}SCA^{+}KIT^{+}$ HPCs after 7 days of E9.5 aggregate culture in the presence of APELIN 13, APELIN 36, or APELA 21 peptides.
(F) Proportion of CD45.2 donor cells present in the peripheral blood of lethally irradiated recipients 14 weeks after transplantation of cells derived from E9 *ex vivo* reaggregate cultures in the absence (0) or presence of 100 or 300 nM APELIN 13, APELIN 36, or APELA 21. Each data point represents one recipient animal.
(G) Proportion of CD45.2 donor cells present in the peripheral blood of lethally irradiated recipients 14 weeks after transplantation of aggregate cultures that consisted of sorted pre-HSC type I and type II cells derived from E11 in coaggregate cultures in the absence (0) or presence of 100 or 300 nM APELIN 13 or APELIN 36.

mL VEGF for 6 days, with a medium change on day 4. At day 6 the cells were harvested using Accutase (Gibco), counted, and single-cell suspensions were stained with antibodies for flow cytometry and/or plated in CFU-C assays that were scored 10 days later. In experiments to assess the effects of activating the APLNR pathway, APELIN peptides were added from day 0, specifically, APELIN36(LVQPRGSRNGPGPWQGGRRKFRRQRPRLSHKGPMPF), its cleaved bioactive pyroglutamyl form (Pyr1), APELIN 13 (QRPRLSHKGPMPF), and APELA 21 (LYRHACPRRRCIPLHSRVPFP) (Phoenix Pharmaceuticals). Macrophage differentiation was carried out as described previously (Haideri et al., 2017).

Human ESC and iPSC Differentiation

Human ESCs and human iPSCs were maintained on CELLstart (Invitrogen)-coated 6-well tissue culture plates (Costar) in StemPro medium comprising DMEM/F12 with GlutaMAX (Invitrogen), 1.8% BSA (Invitrogen), StemPro supplement (Invitrogen), 0.1 mM β-mercaptoethanol (Invitrogen), and 20 ng/mL human bFGF (Invitrogen), as described previously (Jackson et al., 2016; Yang et al., 2017). Cells were passaged mechanically when they reached about 80% confluency using the EZPassage tool (Invitrogen). In experiments to assess the effects of activating the APLNR pathway, APELIN peptides were added from day 0. The use of human ESCs in haematopoietic differentiation experiments was approved by UK Stem Cell Bank.

Generation of Aplnr-tdTomato Reporter ESC Lines

*Aplnr*T2ATdTomato HDR donor vector was based on the backbone pBSK-2A-iCre-frtneofrt (a gift from Heiko Lickert), which was digested with Not1 and EcoR1 to accept two overlapping PCR fragments and a 1 kb 3′ arm by Gibson assembly (NEB).

Aplnr (NM 011784.3) is a single-exon gene and so the coding region of approximately 1 kb acted as the 5′ homology arm. A PCR fragment consisting of the *Aplnr* coding region and the T2A-encoding sequence was generated by amplification of BAC BMQ407G118 using primers overlapping the backbone at the 5′ end and introducing the T2A sequence at the 3′ end. The T2ATdTomato sequence was generated by PCR of pTdTomato (Clontech). The integrity of the vector was tested by sub-cloning the entire expression cassette into CAG-IRES-Puro vector and the tdTomato signal confirmed in transient transfected ESCs by flow cytometry.

Guide RNAs (gRNA) directed to the 3′ end of the *Aplnr* sequence were predicted by the Zhang lab algorithm (https://zlab.bio). gRNAs directed to the sequence 3′ to the *Aplnr* stop codon were identified and tested functionally using the SplitAx assay (Axton et al., 2017). In brief, a SplitAx vector consisting of the sequence encoding the N-terminal of GFP, the *Aplnr* sequence that covered the region that the gRNAs were directed to, and the sequence encoding the C-terminal of GFP that was out of frame with the N-terminal sequence. After transfection of this SplitAx vector and gRNAs into HEK293 cells, gRNAs were selected if they successfully cut the SplitAx vector and resulted in reconstitution of an in-frame GFP. Transfection of gRNA A2 (TGGGTCAGACCCGCTGCACC) binding to CCTGGTGCAGCGGGTCTGACCCA and gRNA B2 (GGAGAAAGTACAGCCATGCT) binding to AGCATGGCTGTACTTTCTCC resulted in a positive GFP signal in the SplitAx assay and were used for the subsequent gene targeting. Guides A2 and B2 were cloned into TOPO blunt after a synthesized U6 promoter (IDT) before sub-cloning into the guide vector, pGL3-U6-sgRNA EGFP (Liu et al., 2018).

Juo9 (subclone of E14) murine ESCs (2×10^6) were transfected in suspension with 3 μg *Aplnr*T2ATdTomato HDR donor vector, 1 μg gRNA-A2 plasmid, 1 μgRNA-B2 plasmid, and 1 μg Cas9 Nickase(D10A)-GFP plasmid (Addgene no. 48140) or Hu Cas9-GFP (Addgene no. 44720) using Xfect stem transfection reagent (Clontech) in 100 μL Xfect buffer and 2 μL Xfect polymer for 4 h. mESC medium was added to a volume of 2 mL and plated out overnight in gelatinized 6-well plates. Following overnight incubation, 40,000 GFP$^+$ cells were FACS sorted and plated in a gelatinized 10 cm plate. G418 selection (400 μg/mL) was added the next day and, after 7 days, 28 G418-resistant colonies from the Cas9 D10A transfections and 80 colonies from the WT Cas9 transfections were generated. Thirty-fve colonies were picked and screened by PCR for correctly targeted 3′ and 5′ ends, which were then confirmed by Southern blot.

Generation of Aplnr-Null ESC Lines

gRNA sequences were identified (http://www.sanger.ac.uk/htgt/wge/find_crisprs). Four gRNAs with PAM sites after the ATG that had a low number of predicted off target binding sites and two guides with the PAM site after the TAA stop codon were selected. Linker nucleotides were added to the ends of the gRNA sequences and both strands synthesized (IDT). gRNAs were annealed and then ligated into the Bbs1 site of linearized pSPCAs9(Guide)-2A-mCherry vector. Combinations of 5′ and 3′ gRNAs were tested by transient transfection of mESCs and screened for excision of the coding region by PCR. Combinations with the highest level of excision in these transient transfections were used to generate clonal *Aplnr*-null ESC. ESCs ($1 \times 10^6 \times 10^{14}$) were transfected with the two appropriate pSPCAs9(Guide)-2A-mCherry vectors using Xfect (Clontech). Forty-eight hours after transfection cells were sorted for high levels of mCherry expression and plated onto 10 cm gelatinized dishes. Ten days later individual colonies were picked and screened using primers that spanned the coding region.

Animals

Staged embryos were obtained by mating C57BL/6 (CD45.2/2), and the morning of discovery of the vaginal plug was designated as day 0.5. For culture and transplantation, embryonic day 9.5 (E9.5) (25–29 sp) and E11.0–E11.5 (>40 sp) samples were used. All experiments with animals were performed under a Project License granted by the Home Office (UK), University of Edinburgh Ethical Review Committee, and conducted in accordance with local guidelines.

Long-Term Repopulation Assay and Blood Chimerism Analysis

CD45.2/2 cells were injected into irradiated 2- to 3-month-old Bl/6J CD45.1/2 heterozygous recipients along with 100,000 CD45.1/1 nucleated bone marrow carrier cells per recipient. Recipients were γ-irradiated using two doses (600 + 550 rad) separated by 3 h. For day 9.5 culture, one embryo equivalent (e.e.) of the 7 day cultured cells was injected. E11 AGM-sorted cells were injected after 5 days of OP9-coaggregate culture at a dose of 1 e.e./recipient.

Donor-derived chimerism was evaluated in peripheral blood at 6 and 14 weeks post transplantation. Erythrocytes were lysed using Pharm Lyse (BD Biosciences), and non-specific binding was blocked with an anti-CD16/32 (Fc-block) followed by staining with anti-CD45.1-APC (clone A20) and anti-CD45.2-PE (clone 104) monoclonal antibodies (eBioscience). Cell populations were identified as CD45.1-PE+ (donor), CD45.1-PE+CD45.2-APC+ (recipient), CD45.1-APC+ (carrier) using FACSCalibur or Fortessa (BD Biosciences).

HSC numbers were assessed using extreme limiting dilution analysis (ELDA) analysis (Hu and Smyth, 2009). Multilineage donor-derived hematopoietic contribution in recipient blood and organs was determined by staining with anti-CD45.1-V450, anti-CD45.2-V500, and lineage-specific anti-Mac1-fluorescein isothiocyanate (FITC), Gr1-PE CD3e-APC, B220-PE-Cy7 monoclonal antibodies (BD Pharmingen).

Ex Vivo Maturation of HSC Precursors

E9.5 and E11.5 embryos were dissected as described (Rybtsov et al., 2014). Tissues were incubated with collagenase/dispase solution (0.12 mg/mL) (Roche) at 37°C as described (Taoudi et al., 2008), washed, and resuspended in PBS (Sigma) containing 3% FCS and then dissociated by pipetting. After dissociation (and sorting) 1 e.e. of the specific cell populations (e.g., type I or type II pre-HSCs) were coaggregated with 10^5 OP9 cells. Five to ten coaggregates (i.e., 5–10 e.e.) per experimental variant were cultured in Iscove's modified Dulbecco's medium (Invitrogen), with 20% of pre-selected, heat-inactivated FCS, L-glutamine, and penicillin/streptomycin supplemented with murine recombinant cytokines (SCF, IL3, and Flt3) each at 100 ng/mL (PeproTech) and various concentrations of APLNR ligand peptides, including APELIN 36 (LVQPRGSRNGPGPWQGGRRKFRRQRPRLSHKGPMPF), its cleaved bioactive pyroglutamyl form (Pyr1) APELIN 13 (QRPRLSHKGPMPF), and APELA 21 (LYRHACPRRRCIPLHSRVPFP) (Phoenix Pharmaceuticals). Coaggregates were cultured on floating 0.8 μm AAWP 25 mm nitrocellulose membranes (Millipore) for 6 days then dissociated using collagenase/dispase as described (Rybtsov et al., 2014).

CFU-C and Endothelial Assays

A methylcellulose-based hematopoietic progenitor assay was routinely used to enumerate hematopoietic progenitors from both mouse (MethoCult GF M3434, STEMCELL Technologies) and human (MethoCult H4434, STEMCELL Technologies) differentiating PSC. Cells were collected from the differentiating PSC culture and prepared in single-cell suspensions. Methylcellulose medium (1.5 mL) was added into a 35 mm low attachment dish. For each cell treatment, two dishes were set up in parallel at densities of 1×10^5 and 5×10^4 for mouse cells and at 1×10^4 and 5×10^3 for human cells, then incubated at 37°C, and then scored between 7–11 and 12–14 days for mouse and human cells, respectively. Colonies were classified based on the morphology using light microscopy. For endothelial assays, cell was placed on an OP9 cell layer in the presence of 50 ng/mL VEGF as described (Rybtsov et al., 2014). After 11 days, cultures were stained with anti-CD31 antibodies to assess endothelial colonies.

FACS and Analysis

Cell suspensions were stained with following antibodies: Ter119-v500 clone (Clone TER-119); anti-CD41-BV421 (brilliant violet 421) or Alexa Fluor 488 (clone MW30reg); anti-CD45 FITC (clone 30-F11); biotinylated anti-VE-cadherin (clone 11.D4.1) followed by incubation with streptavidin-APC (all purchased from BD Pharmingen or BioLegend). Anti-mouse VE-cadherin (VC) antibody was biotinylated in-house using the FluoReporter Mini-Biotin-XX Protein Labeling Kit (Invitrogen). Cell populations were sorted using a FACSAria II sorter (BD) followed by purity checks. Type I pre-HSCs ($Ter119^{-}VC^{+}CD41^{+}CD45^{-}$) and type II pre-HSCs ($Ter119^{-}VC^{+}CD41^{+}CD45^{+}$) were sorted from E11 AGMs as described previously (Rybtsov et al., 2011). Dead cells were excluded using 7AAD staining and fluorescence minus one control staining was used to define gating strategies. Data acquisition and analyses were performed using Fortessa (BD) and FlowJo software.

Single-Cell RNA Sequencing Analysis

The expression profiles of genes encoding APLNR and two if its ligands were analyzed in previously published single-cell RNA sequencing datasets. Specifically, the *Aplnr* expression profile in FACS-sorted $CD31^{+}cKIT^{hi}GATA2^{med}$ cells isolated from the AGM region of E11 embryos was generated using the online browser (https://gottgens-lab.stemcells.cam.ac.uk/DZIERZAK/) (GEO: GSE143637) (Vink et al., 2020). The *APLNR* expression profile in sorted CD34+ cells from the dissected AGM ventral region from CS16 human embryos was analyzed from a GEO downloaded dataset (GEO: GSE1518767; sample GSM4592621) (Crosse et al., 2020). Data were analyzed using the R package Seurat 3.2.2, following the standard pipeline (https://www.cell.com/cell/fulltext/S0092-8674(19)30559-8). In brief, data were normalized using SCTransform, followed by PCA, UMAP, clustering, and TSNE analysis using the first 20 principal components. Expression profiles in iPSC-derived hemato-endothelial cells were obtained from our previously published dataset (EMBL EBI Arrayexpress: E-MTAB-9295) and the plot was generated using our browser available at lab.antonellafidanza.com (Fidanza et al., 2020).

Statistics

Data on histograms presented as average of at least three independent experiments ± SD and difference evaluated using t test or Prism software.

Data and Code Availability

Accession numbers for the published RNA sequencing datasets that were analyzed in this study include GEO: GSE143637 (Vink et al., 2020), GEO: GSE1518767; sample GSM4592621 (Crosse et al., 2020), and EMBL EBI Arrayexpress: E-MTAB-9295 (Fidanza et al., 2020).

SUPPLEMENTAL INFORMATION

Supplemental Information can be found online at https://doi.org/10.1016/j.stemcr.2021.02.003.

AUTHOR CONTRIBUTIONS

M.J., A.H.T., R.A., A.F., S.R., M.K., and S.M. performed the experiments. M.J., A.F., and L.M.F. planned the experiments and wrote the manuscript. A.M. and T.B. provided intellectual input to the execution of the experiments and interpretation of the results.

ACKNOWLEDGMENTS

This work was funded by Bloodwise (to M.J.), the Wellcome Trust (to A.F. and A.H.T.), and the Medical Research Council (to S.R.). We thank Fiona Rossi and Claire Cryer for flow cytometry. The graphical abstract was created using Biorender.com.

Received: June 10, 2020
Revised: February 4, 2021
Accepted: February 5, 2021
Published: March 4, 2021

REFERENCES

Axton, R.A., Haideri, S.S., Lopez-Yrigoyen, M., Taylor, H.A., and Forrester, L.M. (2017). SplitAx: a novel method to assess the function of engineered nucleases. PLoS One *12*, e0171698.

Batsivari, A., Rybtsov, S., Souilhol, C., Binagui-Casas, A., Hills, D., Zhao, S., Travers, P., and Medvinsky, A. (2017). Understanding hematopoietic stem cell development through functional correlation of their proliferative status with the intra-aortic cluster architecture. Stem Cell Reports *8*, 1549–1562.

Bertrand, J.Y., Chi, N.C., Santoso, B., Teng, S., Stainier, D.Y., and Traver, D. (2010). Haematopoietic stem cells derive directly from aortic endothelium during development. Nature *464*, 108–111.

Boisset, J.C., van Cappellen, W., Andrieu-Soler, C., Galjart, N., Dzierzak, E., and Robin, C. (2010). In vivo imaging of haematopoietic cells emerging from the mouse aortic endothelium. Nature *464*, 116–120.

Bruveris, F.F., Ng, E.S., Leitoguinho, A.R., Motazedian, A., Vlahos, K., Sourris, K., Mayberry, R., McDonald, P., Azzola, L., Davidson, N.M., et al. (2020). Human yolk sac-like haematopoiesis generates *RUNX1-*, *GFI1-* and/or *GFI 1B-* dependent blood and SOX17-positive endothelium. Development *147*, dev193037.

Chen, Q., Liu, Y., Jeong, H.W., Stehling, M., Dinh, V.V., Zhou, B., and Adams, R.H. (2019). Apelin(+) endothelial niche cells control hematopoiesis and mediate vascular regeneration after myeloablative injury. Cell Stem Cell *25*, 768–783.e6.

Crosse, E.I., Gordon-Keylock, S., Rybtsov, S., Binagui-Casas, A., Felchle, H., Nnadi, N.C., Kirschner, K., Chandra, T., Tamagno, S., Webb, D.J., et al. (2020). Multi-layered spatial transcriptomics identify secretory factors promoting human hematopoietic stem cell development. Cell Stem Cell *27*, 822–839.e8.

D'Aniello, C., Fiorenzano, A., Iaconis, S., Liguori, G.L., Andolfi, G., Cobellis, G., Fico, A., and Minchiotti, G. (2013). The G-protein-coupled receptor APJ is expressed in the second heart field and regulates Cerberus-Baf60c axis in embryonic stem cell cardiomyogenesis. Cardiovasc. Res. *100*, 95–104.

D'Aniello, C., Lonardo, E., Iaconis, S., Guardiola, O., Liguoro, A.M., Liguori, G.L., Autiero, M., Carmeliet, P., and Minchiotti, G. (2009). G protein-coupled receptor APJ and its ligand apelin act downstream of Cripto to specify embryonic stem cells toward the cardiac lineage through extracellular signal-regulated kinase/p70S6 kinase signaling pathway. Circ. Res. *105*, 231–238.

Dege, C., Fegan, K.H., Creamer, J.P., Berrien-Elliott, M.M., Luff, S.A., Kim, D., Wagner, J.A., Kingsley, P.D., McGrath, K.E., Fehniger, T.A., et al. (2020). Potently cytotoxic natural killer cells initially emerge from erythro-myeloid progenitors during mammalian development. Dev. Cell *53*, 229–239.e7.

Devic, E., Rizzoti, K., Bodin, S., Knibiehler, B., and Audigier, Y. (1999). Amino acid sequence and embryonic expression of msr/apj, the mouse homolog of *Xenopus* X-msr and human APJ. Mech. Dev. *84*, 199–203.

Ditadi, A., Sturgeon, C.M., and Keller, G. (2017). A view of human haematopoietic development from the Petri dish. Nat. Rev. Mol. Cell Biol. *18*, 56–67.

Dzierzak, E., and Medvinsky, A. (2008). The discovery of a source of adult hematopoietic cells in the embryo. Development *135*, 2343–2346.

Fidanza, A., Stumpf, P.S., Ramachandran, P., Tamagno, S., Babtie, A., Lopez-Yrigoyen, M., Taylor, A.H., Easterbrook, J., Henderson, B., Axton, R., et al. (2020). Single cell analyses and machine learning define hematopoietic progenitor and HSC-like cells derived from human PSCs. Blood *136*, 2893–2904.

Haideri, S.S., McKinnon, A., Taylor, A.H., Kirkwood, P., Lewis, P., O'Duibhir, E., Vernay, B., Forbes, S., and Forrester, L.M. (2017). Injection of embryonic stem cell derived macrophages ameliorates fibrosis in a murine model of liver injury. Regenerative Med. *2*, 14.

Hu, Y., and Smyth, G.K. (2009). ELDA: extreme limiting dilution analysis for comparing depleted and enriched populations in stem cell and other assays. J. Immunol. Methods *347*, 70–78. https://doi.org/10.1016/j.jim.2009.06.008.

Huang, C., Jackson, M., Samuel, K., Taylor, A.H., Lowell, S., and Forrester, L.M. (2013). Haematopoietic differentiation is inhibited when Notch activity is enhanced in FLK1(+) mesoderm progenitors. Stem Cell Res. *11*, 1273–1287.

Inui, M., Fukui, A., Ito, Y., and Asashima, M. (2006). Xapelin and Xmsr are required for cardiovascular development in *Xenopus laevis*. Dev. Biol. *298*, 188–200.

Ivanovs, A., Rybtsov, S., Ng, E.S., Stanley, E.G., Elefanty, A.G., and Medvinsky, A. (2017). Human haematopoietic stem cell development: from the embryo to the dish. Development *144*, 2323–2337.

Ivanovs, A., Rybtsov, S., Welch, L., Anderson, R.A., Turner, M.L., and Medvinsky, A. (2011). Highly potent human hematopoietic stem cells first emerge in the intraembryonic aorta-gonad-mesonephros region. J. Exp. Med. *208*, 2417–2427.

Jackson, M., Axton, R.A., Taylor, A.H., Wilson, J.A., Gordon-Keylock, S.A., Kokkaliaris, K.D., Brickman, J.M., Schulz, H., Hummel, O., Hubner, N., et al. (2012). HOXB4 can enhance the differentiation of embryonic stem cells by modulating the hematopoietic niche. Stem Cells *30*, 150–160.

Jackson, M., Ma, R., Taylor, A.H., Axton, R.A., Easterbrook, J., Kydonaki, M., Olivier, E., Marenah, L., Stanley, E.G., Elefanty, A.G., et al. (2016). Enforced expression of HOXB4 in human embryonic stem cells enhances the production of hematopoietic progenitors but

has No effect on the maturation of red blood cells. Stem Cells Transl. Med. *5*, 981–990.

Kidoya, H., Naito, H., Muramatsu, F., Yamakawa, D., Jia, W., Ikawa, M., Sonobe, T., Tsuchimochi, H., Shirai, M., Adams, R.H., et al. (2015). APJ regulates parallel alignment of arteries and veins in the skin. Dev. Cell *33*, 247–259.

Kidoya, H., Naito, H., and Takakura, N. (2010). Apelin induces enlarged and nonleaky blood vessels for functional recovery from ischemia. Blood *115*, 3166–3174.

Kidoya, H., Ueno, M., Yamada, Y., Mochizuki, N., Nakata, M., Yano, T., Fujii, R., and Takakura, N. (2008). Spatial and temporal role of the apelin/APJ system in the caliber size regulation of blood vessels during angiogenesis. EMBO J. *27*, 522–534.

Kissa, K., and Herbomel, P. (2010). Blood stem cells emerge from aortic endothelium by a novel type of cell transition. Nature *464*, 112–115.

Liu, Z., Lu, Z., Yang, G., Huang, S., Li, G., Feng, S., Liu, Y., Li, J., Yu, W., Zhang, Y., et al. (2018). Efficient generation of mouse models of human diseases via ABE- and BE-mediated base editing. Nat. Commun. *9*, 2338.

Malaguti, M., Nistor, P.A., Blin, G., Pegg, A., Zhou, X., and Lowell, S. (2013). Bone morphogenic protein signalling suppresses differentiation of pluripotent cells by maintaining expression of E-Cadherin. eLife *2*, e01197.

McGrath, K.E., Frame, J.M., Fegan, K.H., Bowen, J.R., Conway, S.J., Catherman, S.C., Kingsley, P.D., Koniski, A.D., and Palis, J. (2015). Distinct sources of hematopoietic progenitors emerge before HSCs and provide functional blood cells in the mammalian embryo. Cell Rep. *11*, 1892–1904.

Medvinsky, A., and Dzierzak, E. (1996). Definitive hematopoiesis is autonomously initiated by the AGM region. Cell *86*, 897–906.

Medvinsky, A., Taoudi, S., Mendes, S., and Dzierzak, E. (2008). Analysis and manipulation of hematopoietic progenitor and stem cells from murine embryonic tissues. Curr. Protoc. Stem Cell Biol. *Chapter 2*, Unit 2A 6.

Medvinsky, A.L., and Dzierzak, E.A. (1998). Development of the definitive hematopoietic hierarchy in the mouse. Dev. Comp. Immunol. *22*, 289–301.

Oatley, M., Bölükbası, Ö., Svensson, V., Shvartsman, M., Ganter, K., Zirngibl, K., Pavlovich, P.V., Milchevskaya, V., Foteva, V., Natarajan, K.N., et al. (2020). Single-cell transcriptomics identifies CD44 as a marker and regulator of endothelial to haematopoietic transition. Nat. Commun. *11*, 586.

Palis, J. (2014). Primitive and definitive erythropoiesis in mammals. Front. Physiol. *5*, 3.

Quertermous, T. (2007). Apelin and its G protein-coupled receptor regulate cardiac development as well as cardiac function. Dev. Cell *12*, 319–320.

Rybtsov, S., Batsivari, A., Bilotkach, K., Paruzina, D., Senserrich, J., Nerushev, O., and Medvinsky, A. (2014). Tracing the origin of the HSC hierarchy reveals an SCF-dependent, IL-3-independent CD43(–) embryonic precursor. Stem Cell Reports *3*, 489–501.

Rybtsov, S., Sobiesiak, M., Taoudi, S., Souilhol, C., Senserrich, J., Liakhovitskaia, A., Ivanovs, A., Frampton, J., Zhao, S., and Medvinsky, A. (2011). Hierarchical organization and early hematopoietic specification of the developing HSC lineage in the AGM region. J. Exp. Med. *208*, 1305–1315.

Scott, I.C., Masri, B., D'Amico, L.A., Jin, S.W., Jungblut, B., Wehman, A.M., Baier, H., Audigier, Y., and Stainier, D.Y. (2007). The G protein-coupled receptor agtrl1b regulates early development of myocardial progenitors. Dev. Cell *12*, 403–413.

Souilhol, C., Gonneau, C., Lendinez, J.G., Batsivari, A., Rybtsov, S., Wilson, H., Morgado-Palacin, L., Hills, D., Taoudi, S., Antonchuk, J., et al. (2016). Inductive interactions mediated by interplay of asymmetric signalling underlie development of adult haematopoietic stem cells. Nat. Commun. *7*, 10784.

Sugimura, R., Jha, D.K., Han, A., Soria-Valles, C., da Rocha, E.L., Lu, Y.F., Goettel, J.A., Serrao, E., Rowe, R.G., Malleshaiah, M., et al. (2017). Haematopoietic stem and progenitor cells from human pluripotent stem cells. Nature *545*, 432–438.

Suzuki, N., Yamazaki, S., Yamaguchi, T., Okabe, M., Masaki, H., Takaki, S., Otsu, M., and Nakauchi, H. (2013). Generation of engraftable hematopoietic stem cells from induced pluripotent stem cells by way of teratoma formation. Mol. Ther. *21*, 1424–1431.

Taoudi, S., Gonneau, C., Moore, K., Sheridan, J.M., Blackburn, C.C., Taylor, E., and Medvinsky, A. (2008). Extensive hematopoietic stem cell generation in the AGM region via maturation of VE-cadherin+CD45+ pre-definitive HSCs. Cell Stem Cell *3*, 99–108.

Taoudi, S., and Medvinsky, A. (2007). Functional identification of the hematopoietic stem cell niche in the ventral domain of the embryonic dorsal aorta. Proc. Natl. Acad. Sci. U S A *104*, 9399–9403.

Taoudi, S., Morrison, A.M., Inoue, H., Gribi, R., Ure, J., and Medvinsky, A. (2005). Progressive divergence of definitive haematopoietic stem cells from the endothelial compartment does not depend on contact with the foetal liver. Development *132*, 4179–4191.

Vink, C.S., Calero-Nieto, F.J., Wang, X., Maglitto, A., Mariani, S.A., Jawaid, W., Göttgens, B., and Dzierzak, E. (2020). Iterative single-cell analyses define the transcriptome of the first functional hematopoietic stem cells. Cell Rep. *31*, 107627.

Vodyanik, M.A., Yu, J., Zhang, X., Tian, S., Stewart, R., Thomson, J.A., and Slukvin, I.I. (2010). A mesoderm-derived precursor for mesenchymal stem and endothelial cells. Cell Stem Cell *7*, 718–729.

Yang, C.T., Ma, R., Axton, R.A., Jackson, M., Taylor, A.H., Fidanza, A., Marenah, L., Frayne, J., Mountford, J.C., and Forrester, L.M. (2017). Activation of KLF1 enhances the differentiation and maturation of red blood cells from human pluripotent stem cells. Stem Cells *35*, 886–897.

Yu, Q.C., Hirst, C.E., Costa, M., Ng, E.S., Schiesser, J.V., Gertow, K., Stanley, E.G., and Elefanty, A.G. (2012). APELIN promotes hematopoiesis from human embryonic stem cells. Blood *119*, 6243–6254.

Stem Cell Reports, Volume *16*

Supplemental Information

Modulation of APLNR Signaling Is Required during the Development and Maintenance of the Hematopoietic System

Melany Jackson, Antonella Fidanza, A. Helen Taylor, Stanislav Rybtsov, Richard Axton, Maria Kydonaki, Stephen Meek, Tom Burdon, Alexander Medvinsky, and Lesley M. Forrester

Jackson et al Supplementary Figures

Supplementary Figure S1. Production of *Aplnr*-tdTomato reporter ESC line.

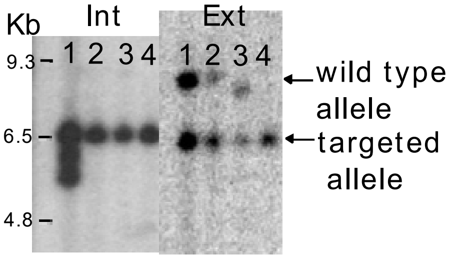

Southern blotting screening of clones from *Aplnr*-tdTomato reporter ESC line production. Genomic DNA of G418-resistant clones was performed using internal (int) and external probe to the Neo sequence. Clones 2, 3 and 4 demonstrated a single integration of the targeting vector into the correct site using the internal probe to the neo gene.

Supplementary Figure S2. Commercial α-APLNR antibody is not specific for APLNR.

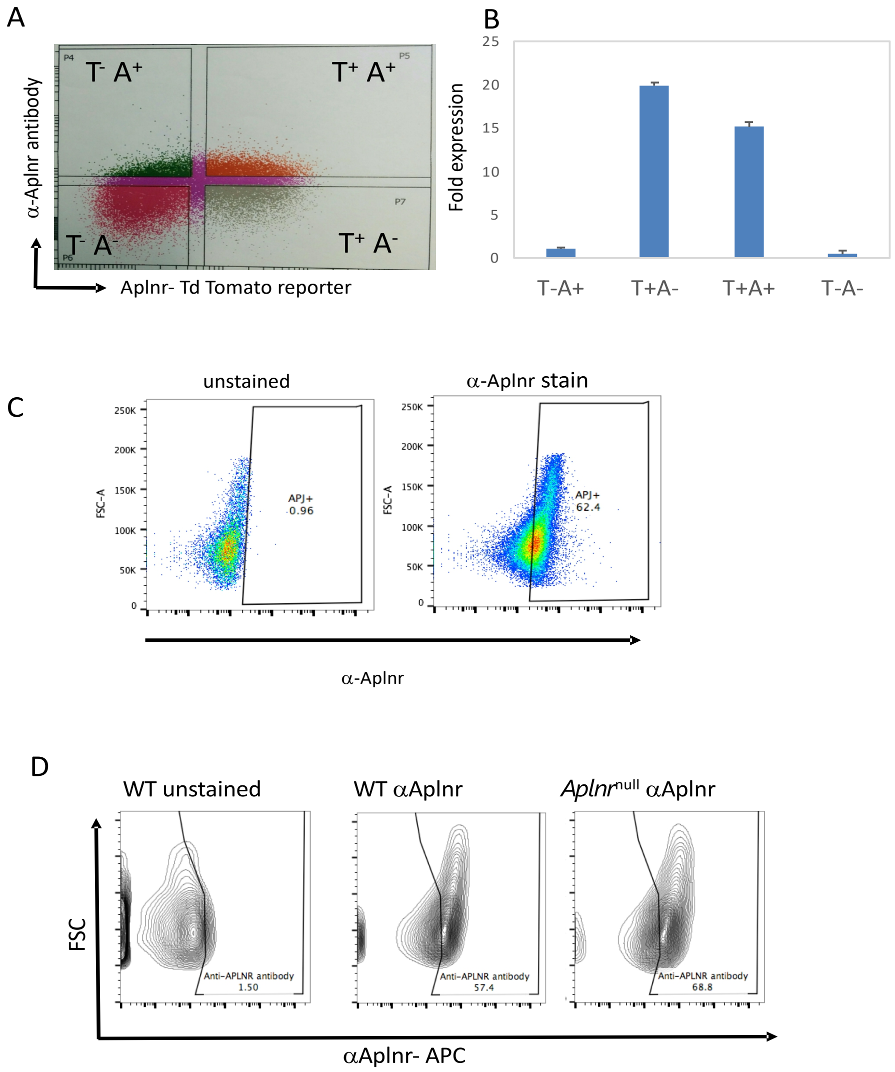

A. Flow cytometry analyses of the *Aplnr*-tdTomato reporter ESC line stained with a commercial antibody to APLNR.
B. qRT-PCR analyses of FAC-sorted cells based on the expression of the tdTomato reporter (T) and α-APLNR antibody staining (A). The presence of *Aplnr* transcripts correlate with the tdTomato expression but not with cells identified by the α-APLNR antibody.
C. Control flow cytometry analyses of 293T cells stained with the α-APLNR antibody demonstrates the poor specificity of the α-APLNR antibody as the 293T cell line does not express *Aplnr* transcripts.
D. Flow cytometry analyses of control, wild type (WT) ESCs and *Aplnr*-null ESCs stained with the anti-APLNR antibody and unstained control.

Supplementary Figure S3. Gating strategy for *Aplnr*-td-Tomato-expressing cells

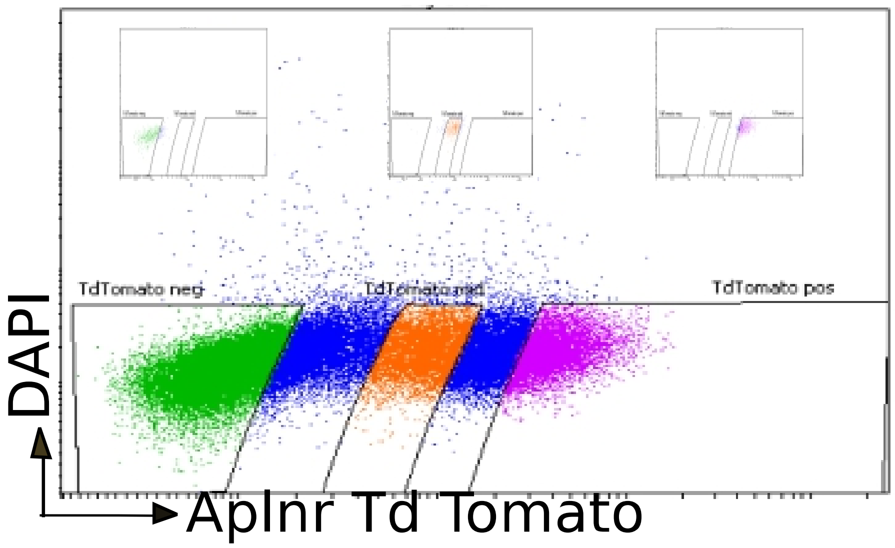

Flow cytometry plot demonstrating the gating for sorting of *Aplnr*-tdTomato high , *Aplnr*-tdTomato intermediate and *Aplnr*-tdTomato negative cell populations. Insert show the re-analysis of sorting cells demonstrating purity of each of the populations.

Supplementary Figure S4. Generation of *Aplnr*-null ESC line.

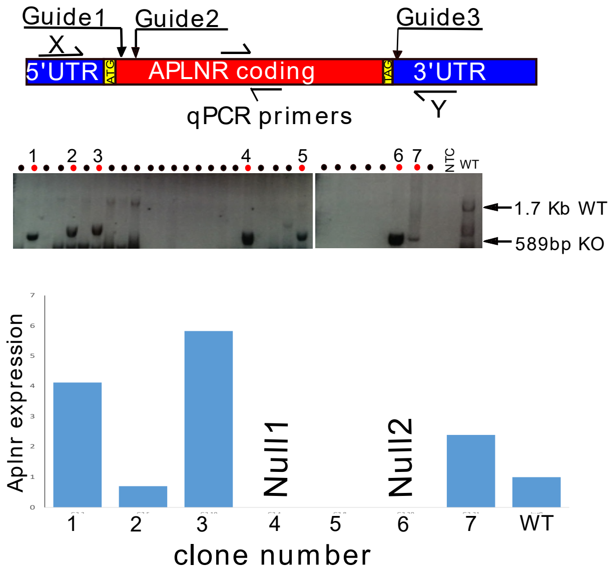

A. Schematic of Crispr/Cas9 strategy to knockout the *Aplnr* coding region showing guide RNAs (Guide 1, Guide 2) and primers that were used to genotype resultant ESC clones and to validate depletion of *Aplnr* transcripts in qRT- PCR experiments.
B. Southern blot showing the presence of the wild type, 1700 base pair restriction fragment in control ESC and the predicted 589 base pair fragment in targeted clones.
C. qRT-PCR of the parental control ESC line (WT) and individual targeted ESC clones (1-7) showing that 2 of these (clones 4 and 6) had no *Aplnr* transcripts, confirming functional homozygosity. These were then defined as Null 1 and Null 2, respectively.

Supplementary Figure S5. Expression of *Aplnr* mRNA transcripts and two potential Apelin and Apela transcripts during mouse ESC differentiation.

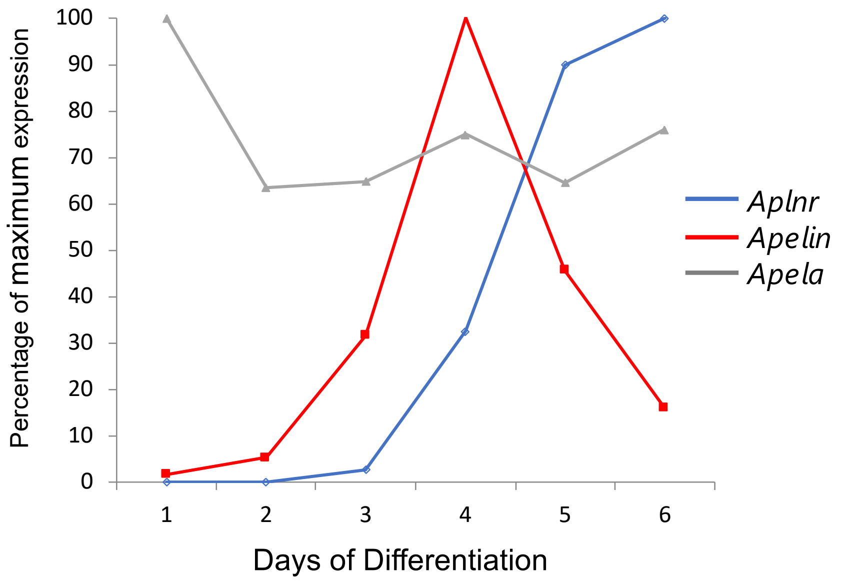

Quantitative RT-PCR analyses of cells undifferentiated mouse ESC (day 0) and at subsequent stages of differentiation in the presence of serum.

Supplementary Figure S6. Addition of APELIN ligands to CFU-C assays increases the number of CFU-M colonies generated from differentiating human PSCs.

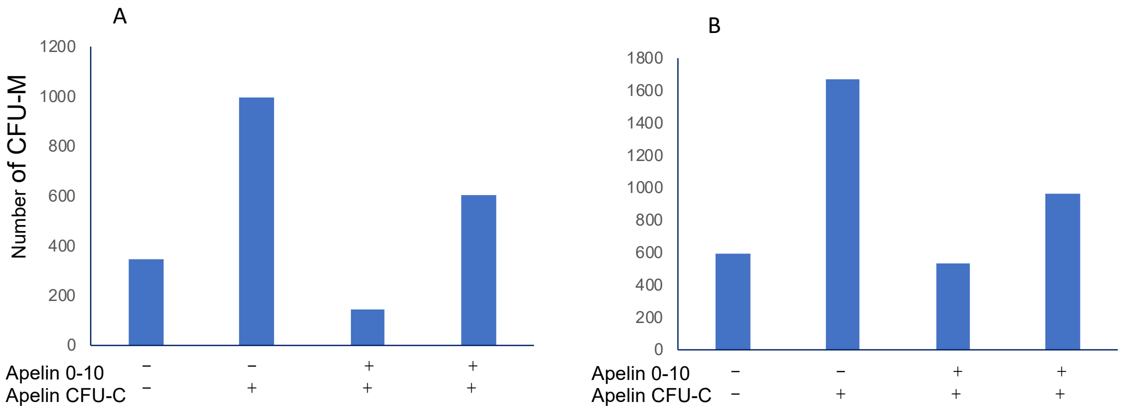

Human H1 ESCs (A) and SFCi55 iPSCs (B) were differentiated for 10 days without APELIN 36 (-) or with the addition of 300 nM APELIN 36 (+) at day 2, 4 and 7 then cells were disaggregated and plated in methylcellulose cultures for a further 10 days in the presence (+) or absence (-) of 300nM APELIN 36 (n=1)
